# Pathogenic Determinants of the *Mycobacterium kansasii* Complex: An Unsuspected Role for Distributive Conjugal Transfer

**DOI:** 10.3390/microorganisms9020348

**Published:** 2021-02-10

**Authors:** Florian Tagini, Trestan Pillonel, Claire Bertelli, Katia Jaton, Gilbert Greub

**Affiliations:** 1 Department of Laboratory Medicine, Institute of Microbiology, Lausanne University Hospital and University of Lausanne, 1011 Lausanne, Switzerland; florian.tagini@chuv.ch (F.T.); trestan.pillonel@chuv.ch (T.P.); claire.bertelli@chuv.ch (C.B.); katia.jaton-ogay@chuv.ch (K.J.); 2 Division of Infectious Diseases, Department of Medicine, Lausanne University Hospital, 1011 Lausanne, Switzerland

**Keywords:** whole-genome sequencing, virulence, virulence factor, nontuberculous mycobacteria, genomics, conjugative plasmid, horizontal gene transfer, conjugation, transduction, mosaicism

## Abstract

The *Mycobacterium kansasii* species comprises six subtypes that were recently classified into six closely related species; *Mycobacterium kansasii* (formerly *M. kansasii* subtype 1), *Mycobacterium persicum* (subtype 2), *Mycobacterium pseudokansasii* (subtype 3), *Mycobacterium ostraviense* (subtype 4), *Mycobacterium innocens* (subtype 5) and *Mycobacterium attenuatum* (subtype 6). Together with *Mycobacterium gastri*, they form the *M. kansasii* complex. *M. kansasii* is the most frequent and most pathogenic species of the complex. *M. persicum* is classically associated with diseases in immunosuppressed patients, and the other species are mostly colonizers, and are only very rarely reported in ill patients. Comparative genomics was used to assess the genetic determinants leading to the pathogenicity of members of the *M. kansasii* complex. The genomes of 51 isolates collected from patients with and without disease were sequenced and compared with 24 publicly available genomes. The pathogenicity of each isolate was determined based on the clinical records or public metadata. A comparative genomic analysis showed that all *M. persicum*, *M. ostraviense*, *M innocens* and *M. gastri* isolates lacked the ESX-1-associated EspACD locus that is thought to play a crucial role in the pathogenicity of *M. tuberculosis* and other non-tuberculous mycobacteria. Furthermore, *M. kansasii* was the only species exhibiting a 25-Kb-large genomic island encoding for 17 type-VII secretion system-associated proteins. Finally, a genome-wide association analysis revealed that two consecutive genes encoding a hemerythrin-like protein and a nitroreductase-like protein were significantly associated with pathogenicity. These two genes may be involved in the resistance to reactive oxygen and nitrogen species, a required mechanism for the intracellular survival of bacteria. Three non-pathogenic *M. kansasii* lacked these genes likely due to two distinct distributive conjugal transfers (DCTs) between *M. attenuatum* and *M. kansasii*, and one DCT between *M. persicum* and *M. kansasii*. To our knowledge, this is the first study linking DCT to reduced pathogenicity.

## 1. Introduction

*Mycobacterium kansasii*, a member of non-tuberculous mycobacteria (NTM), is an environmental bacterium that can cause pulmonary diseases mostly in patients with immunosuppression or underlying lung diseases. Although it depends on the geographical area, *M. kansasii* is usually classed as the second or third most common NTM isolated from patients [1,2]. The diagnosis of pulmonary *M. kansasii* infections relies on clinical, radiological and microbiological findings [3]. Clinical findings include chronic cough, sputum production, fatigue, fever, hemoptysis, chest pain and weight loss. Chest X-ray and CT-Scan classically show cavitation, nodules or bronchiectasis. Finally, microbiological criteria for the diagnosis are (i) two positive cultures from two separated sputum samples, or (ii) positive culture from one bronchoalveolar lavage (BAL), or (iii) mycobacterial histopathological findings on the lung biopsies and one positive culture (of sputum or BAL). In addition, *M. kansasii* can also cause skin, soft tissues, and osteoarticular infections in both immunocompetent and immunocompromized patients [4]. In some very sporadic cases, *M. kansasii* can also be found in the urine [5], sometimes with pathogenic manifestations [6].

*M. kansasii* isolates was previously classified into seven different subtypes, based on PCR and restriction fragment length polymorphisms (RFLP) of the gene *hsp65* [7,8]. Discrimination using other typing schemes, offering the advantage of using only one restriction enzyme, have also been proposed [9]. A recent whole-genome sequence analysis showed that each subtype, except subtype 7 (not available for the study), corresponds to new species-level lineages of the *M. kansasii* complex [10]. The *M. kansasii* species is now restricted to subtype 1, which includes the most common and pathogenic strains for both immunocompetent and immunocompromised patients [11]. Conversely, subtype 2, renamed *M. persicum*, is found mainly in HIV^+^ patients [10,12]. The four other subtypes—subtype 3, 4, 5 and 6, reclassified as *M. pseudokansasii*, *M. ostraviense*, *M. innocens* and *M. attenuatum*, respectively—are rarely associated with diseases and mostly remain colonizers [10,13].

*M. kansasii* shares many virulence factors with *M. tuberculosis*. For instance, *M. kansasii* encodes the type-VII secretion system ESX-1 locus, involved in secretion of several effectors like EsxA (ESAT-6) and EsxB (CFP-10), through its ESX conserved components (called Ecc proteins) [14]. ESX-1-secreted proteins are thought to modulate the activation of macrophages, allow phagosomal escape and induce necrosis/apoptosis [15]. EsxA is a helix-turn-helix hairpin 6 kDa protein, whose length matchds the thickness of eukaryotic cells’ membranes, and might form insertions that could disrupt the phagosomal membranes [16]. In addition, another operon encoding the ESX-1-secreted effectors EspA, EspC and EspD was reported in *M. kansasii* [14] and is absent from non-pathogenic slow-growing mycobacteria [15]. The *espACD* operon was thus hypothesized to be a mycobacterial pathogenicity island [17,18]. Secretion of EspA and EspC is co-dependent on the reciprocal secretion of EsxA and EsxB, supporting the role of the *espACD* operon in ESX-1 function [19]. In vivo experiments and Phagosomal Perturbation Assay in THP-1 macrophages showed that *M. kansasii* can disrupt the phagosomal membrane and exhibited similar bacterial loads as *M. tuberculosis* [14]. Phagosomal disruption and translocation to the cytosol, a crucial virulence mechanism, was shown to occur for a *M. kansasii* strain and not for an environmental *M. innocens* strain [20]. *M. kansasii* escape was further confirmed by phagosomal perturbation FRET-based assays [14]. In addition, *M. kansasii* strains produced higher amounts of Esx-B (CFP-10) than *M. pseudokansasii* and *M. ostraviense* strains [21]. However, these results were obtained by Western blot from culture medium and could have been influenced by (i) the growth phase that could affect the production of Esx-B and (ii) the use of monoclonal antibodies despite protein sequences are different between subtypes. Finally, pathogenic *M. kansasii* and *M. pseudokansasii* strains were able to grow better than non-pathogenic *M. pseudokansasii* and *M. attenuatum* in *Acanthamoeba castellanii*, suggesting an increased resistance to phagocytic cells [22].

Transformation, transduction and conjugation are the three primary mechanisms of horizontal gene transfer (HGT) in bacteria [23]. There is currently no evidence for recent or ongoing HGTs in members of the *M. tuberculosis* complex, except *M. canettii* [24,25]. However, HGTs occur widely in non-tuberculous mycobacteria. Natural transformation, characterized by the horizontal acquisition of usually small DNA fragments (3–5 Kb) by an active bacterial uptake, was reported in *M. smegmatis* and *M. avium* [26,27,28]. Transduction, mediated by bacteriophages, also occurs in the *Mycobacterium* genus [29,30]. Unlike conjugation, these two mechanisms do not require cell-to-cell contacts to transfer DNA. Conjugative plasmids have been described in *M. marinum*, *M. kansasii*, *M. avium* and *M. yongonense*, and encode both type-IV and type-VII secretion systems loci [31]. A plasmid conjugation event between *M. avium* and *M. kansasii* within a patient with disseminated mixed infection was recently reported and confirmed experimentally [32]. These plasmids can carry a very diverse set of genes, but there is currently no evidence for a role in the pathogenicity of NTM [14,31]. To our knowledge, the only plasmid associated with virulence in mycobacteria is the non-conjugative plasmid found in *M. ulcerans*, the causative agent of Buruli ulcers [33]. Chromosomal transfers were reported decades ago in *M. smegmatis* [34], but were only recently better characterized as a powerful conjugative mechanism called distributive conjugal transfer (DCT) [35]. DCT occurs frequently among mycobacteria, transferring DNA from a donor cell to a recipient cell through a process involving the type-VII secretion system ESX-1 and ESX-4 [28]. Genomic DNA fragments of the donor cell ranging from 59 base pairs (bp) to more than 200 Kbp can be integrated into the recipient cell by homologous recombination and generate both macro- and micro-mosaic genomes [28]. DCT drastically differs from the Hfr conjugation model, where a conjugative plasmid is integrated in the chromosome [36]. Hfr conjugal events can lead to transfers of genomic regions downstream of the integrated episome, but do no produce mosaic genomes as seen with DCT.

To test the hypothesis that pathogenicity in the *M. kansasii* complex is based on genetic determinants carved by evolutionary processes, we performed a comparative genome analysis of 75 strains with varying level of pathogenicity. Genetic variations, including single nucleotide variants and gene gain/loss, were associated with pathogenicity, and shaped by intra- and inter-species HGT events that occurred by distributive conjugal transfer.

## 2. Materials and Methods

### 2.1. Strain Inclusion and Pathogenicity Classification

This study included all strains belonging to the *M. kansasii* complex isolated from patients between 2007 and 2017 at the Lausanne University Hospital, a Swiss tertiary care hospital. Strains were classified into five different categories: 1, pathogenic; 2, probably pathogenic; 3, unknown; 4, probably non-pathogenic/colonizer; 5, non-pathogenic/colonizer. The presence of a mycobacterial disease and its compliance with 2007′s criteria of the American Thoracic Society (ATS) for pulmonary NTM diseases was assessed based on the available clinical data [3]. “Probably pathogenic” cases did not fulfil completely all the ATS criteria, but the pathogenicity was strongly suspected based on available clinical, microbiological and/or radiological data. “Probably non-pathogenic/colonizer” cases were cases for which clinical, microbiological and/or radiological data indicated that the strain was likely a colonizer, but for which detailed data were not available. In our cohort, osteoarticular manifestations of *M. kansasii* complex diseases were all considered pathogenic (category 1). Proportions of pathogenic strains in the different species were compared using the “N-1” chi-squared test [37].

This dataset was completed with strains from two different external sources. Five *M. kansasii* and six *M. pseudokansasii* strains available from Goy et al. [22] were included. The pathogenicity of those pulmonary isolates had already been determined according to the 1997′s criteria of the ATS for mycobacterial diseases [11,38]. This study also included a single *M. pseudokansasii* strain (MK142) that was isolated from blood culture of a patient with disseminated mycobacterial infection and thus classified as pathogenic [22]. Moreover, all publicly available genomes identified as *M. kansasii* and *M. gastri* in the NCBI Taxonomy database were used for the comparative genome analysis (as available in June 2017). The pathogenicity of these isolates was determined based on available metadata and then determined as either “unknown”, “probably pathogenic” or “probably non-pathogenic/colonizer”. The genome of *M. tuberculosis* reference strain H37Rv (GCF_000195955.2) was added to the dataset to perform genomic comparisons and to root the phylogenetic tree of NTM strains.

### 2.2. Bacterial Cultures, DNA Extraction and Sequencing

Mycobacteria were grown in Mycobacterial Growth Indicator Tubes BD BACTEC™ MGIT™, supplemented with MGIT™ OADC enrichment and MGIT™ PANTA™ antibiotic mixture (Becton Dickinson, Franklin Lakes, NJ, USA). DNA was extracted using the Wizard Genomic DNA Purification System (Promega, Madison, WI, USA, ref. A1120), following the protocol for mycobacteria. Briefly, culture pellets were inactivated for 1 h at 95 °C. Pellets were then resuspended in a solution of 300 μL of 50 mM EDTA pH 8.0 and 50 μL lysozyme 100 mg/mL, and incubated for 1 h at 37 °C. After 2 min of centrifugation at 20,000× *g*, supernatants were resuspended in solution of 500 μL of Nuclei lysis, 100 μL SDS 20%, 150 μL EDTA 0.5 M pH 8, 20 μL Proteinase K 20 mg/mL and 3 μL RNAse A and incubated for (i) 1 h at 55 °C and (ii) for 5 min at 70 °C. Approximately 150 μL of microbeads <106 μm (Sigma, Saint-Louis, MO, USA, ref. G4649) were added before shaking the samples with FastPrep at 6800 rpm for 3 × 30 s. Samples were centrifuged for 2 min at 20,000× *g* before transferring the supernatant into a new tube. The protocol for bacteria was then continued (200 μL protein precipitation solution, precipitation step with isopropanol 100% and washing steps with ethanol 70%). For MiSeq sequencing (Illumina, San Diego, CA, USA), final elution was done in 30 μL of 10 mM TRIS/0.1 mM EDTA pH 8.0 and incubated for 1 h at 65 °C. For Pacific Biosciences RS II sequencing (Menlo Park, CA, USA), elution was done in 30 μL of 10 mM TRIS pH 8.5 and incubated overnight at 4 °C.

Genomic libraries were prepared using Nextera XT library kit (Illumina, San Diego, CA, USA, ref. FC-131–1096) and size distributions were checked using an automated capillary electrophoresis instrument (Fragment Analyzer™ Automated CE System, Advanced Analytical, Agilent Technologies, Santa Clara, CA, USA). Whole genome sequencing was performed with a MiSeq sequencer (Illumina, San Diego, CA, USA) using either 150 bp or 250 bp paired-end reads. Strain MK142 was sequenced with a Pacific Biosciences RS II sequencer using one SMRT cell version P6-C4 (Pacific Biosciences, Menlo Park, CA, USA) as described in Tagini et al. [10].

### 2.3. Quality Controls, Assembly, Annotation and Comparative Genomic Analyses

Read quality was assessed with FastQC v0.11.4 before and after trimming (Andrews S, available online at: http://www.bioinformatics.babraham.ac.uk/projects/fastqc, accessed on 8 February 2021). Reads were trimmed with Trimmomatic v0.35 (MINLEN:60, LEADING:9, TRAILING:9, SLIDING-WINDOW:3:15) [39]. Assemblies were performed using SPAdes v3.9.0 [40] with k-mer sizes ranging from 41 to 127 bp. Contigs were reordered with Mauve software 13 February 2015 snapshot for Linux using *M. kansasii* ATCC 12478 as a reference [41]. Genomes were annotated using Prokka 1.12 [42].

For the comparative genomic analyses, a MySQL database was created as described in Tagini et al. to perform comparative analyses and browse gene annotation using a web interface [43]. Briefly, each coding sequence was BLASTed (using BLASTP algorithm) against the protein Cluster of Orthologous Genes (COG) database with e-value, amino acid identity and query coverage cut-offs of 10^−5^, 20% and 50%, respectively [44,45]. Kegg Orthology (KO) numbers were assigned using GhostKOALA v2.0 [46]. Protein domains were predicted using InterProScan v5.18-57.0 [47]. Orthologous and paralogous sequences were identified and clustered into so-called “orthogroups” using OrthoFinder v1.1.4 [48]. For each orthogroup, the amino acid sequences were aligned using Mafft v7.187 [49], and a maximum-likelihood phylogeny was built using RaxML v8.2.10 with parameters “-m PROTGAMMALG –p 12345” and “–f J” to compute the node supports [50]. Finally, available gene operons data were downloaded from the DOOR 2.0 database [51].

Multiple sequence alignments of single copy orthogroups conserved in all strains were concatenated into a single amino acid alignment that was used to reconstruct a maximum-likelihood phylogeny using FastTree v2.1.8 with parameters “–gamma –spr 4—mlacc 2–slownni” [52]. This phylogeny, the matrix of orthogroup presence/absence (including plasmid-encoded genes) as well as the degree of pathogenicity were used as input of a genome-wide association study (GWAS) on the whole dataset performed with TreeWAS v1.0 [53]. Furthermore, all genomes of the species *M. kansasii* were aligned using ParSNP v1.2 to identify conserved genomic regions (or core *M. kansasii* genome). Strains from other species were excluded to maximize the size of the core-genome alignment [54]. Then, trimmed reads of *M. kansasii* isolates were mapped onto identified conserved regions of strain ATCC 12478 using Snippy v3.1 [55]. A joint matrix of SNP presence/absence as well as single-copy orthogroups was built to test their association with the degree of pathogenicity and tissue tropism of *M. kansasii* strains only using TreeWAS v1.0 [53].

The presence of homologs to *M. tuberculosis* virulence factors in the orthogroups was assessed using the locus tags of (1) the genes listed in Forrellad et al. [56] and (2) the genes related to type-VII secretion systems loci (ESX-1, EspACD, ESX-2, ESX-3, ESX-4 and ESX-5) described in Simeone et al. [57]. Furthermore, genes specific to each species and each clinical presentation were identified and their annotation manually inspected to assess the presence of genes that could be involved in virulence.

### 2.4. Detection of Distributive Conjugal Transfers between Members of the M. kansasii Complex

Distributive conjugal transfers (DCTs) between strains of different species of the *M. kansasii* complex were identified by looking for genes exhibiting higher similarity between strains of different species than among strains of the same species. Practically, the mean amino acid identity with strains of the same species and with strains of the six other species of the *M. kansasii* complex was calculated for each locus. In the case of one-to-many orthology relationships, the amino acid identity of the closest orthologous gene was used for calculation. Then, if the mean amino acid identity with strains of another species was higher than with strains of the same species, the locus was considered as part of a candidate DCT region involving recombination between the considered strain and other species. Regions containing prophages were detected using the PHAge Search Tool Enhanced Release (PHASTER) [58,59]. All DCTs were represented using genoPlotR v0.8.7 [60].

### 2.5. Detection of Conjugative Plasmids

To detect conjugative plasmids in newly sequenced and publicly available draft genomes, complete plasmid sequences of *M. kansasii* pMK12478 and *M. pseudokansasii* pMK142 were used to perform BLASTN searches against the draft genomes (contig files) of the dataset with an e-value cutoff of 0.00001. Contigs were extracted when returning more than one hit or if the alignment length exceeded 1000 bp. In addition, potential new conjugative plasmids were identified by searching for highly conserved genes of the mycobacterial conjugative machinery [31]. Contigs encoding homologs of the relaxase of pMK12478 (MKAN_RS28485) and VirB4 (MKAN_RS28085) were identified based on OrthoFinder grouping. In addition, open reading frames that could have been missed by the automated annotation were searched in contigs with TBLASTN. Since conjugative plasmids encode an additional type-VII secretion system locus, Hidden-Markov Model (HMM) searches for the ESX conserved components proteins and WxG100 effector proteins (like EsxA and EsxB) were performed using MacSyFinder v.1.0.4 with a custom database of published HMM models (TIGR03922, EccA; TIGR03919, EccB; TIGR03924, EccCa; TIGR03925, EccCb; TIGR03920, EccD; TIGR03923, EccE; TIGR03921, MycP; TIGR03930, WxG100 effector proteins) [61]. Variations in contig GC content and sequencing depth were investigated to identify contigs exhibiting similar characteristics as candidate plasmids sequences identified by homology. Only contigs encoding at least the extra type-VII secretion system plus a relaxase or a VirB4 homolog were considered in the comparative analysis. Episomes integrated in the middle of large contigs were not excluded. All plasmids were reannotated using Prokka v1.12 [42]. A MySQL plasmid database was created as described above with all detected conjugative plasmids as well as pRAW, a conjugative plasmid of *M. marinum* described by Ummels et al. that shares a common backbone structure with the plasmid of *M. kansasii* strain ATCC 12478 [31].

## 3. Results

### 3.1. M. kansasii Is More Pathogenic than Other Members of the Complex

Among the 40 strains isolated at the Lausanne University Hospital between 2007 and 2017, seventeen were isolated from patients presenting non-tuberculosis mycobacterial diseases (category 1 or 2) (Table 1). An immunosuppression was documented in seven of them. As expected, most patients with diseases had pulmonary clinical presentations. However, five bone and joint infections and one urinary tract infection were also reported, hence representing 35% of diseased patients. For nine patients, the strain pathogenicity could not be assessed due to the lack of clinical data. Finally, fourteen patients were only colonized with species of the *M. kansasii* complex (category 4 or 5), although two of them were immunosuppressed. MK8 and MK11, both *M. pseudokansasii*, were the only bacteria isolated from urine.

We compared the proportion of pathogenic/probably pathogenic strains between the most frequently isolated species, *M. kansasii*, *M. persicum* and *M. pseudokansasii* (Table 1). For the calculation, only strains recovered between 2007 and 2017 in the University hospital of Lausanne were used to avoid a selection bias (published genomes tend to be sequenced from more pathogenic strains). Strains with undetermined pathogenicity were excluded from the calculations. The proportions of pathogenic/probably pathogenic strains were 72%, 60% and 14% for *M. kansasii* (total = 18), *M. persicum* (total = 5) and *M. pseudokansasii* (total = 7), respectively. The difference was only statistically significant between *M. kansasii* and *M. pseudokansasii* (*p*-value of 0.01) likely because of the small sample size of *M. persicum*. These findings are congruent with a previous report [11].

It is interesting to note that MK48 and MK54 were recovered from two patients of the same family. However, transmission between patients or transmission from the same contaminated environment can be ruled out since MK48 strain clustered with *M. pseudokansasii* and MK54 with *M. persicum* strains. A familial genetic predisposition or predisposing disease (one of them was known for bronchiectasis) is thus the most likely hypothesis.

### 3.2. Genomic Features and Core-Genome Phylogeny

**Figure 1:**
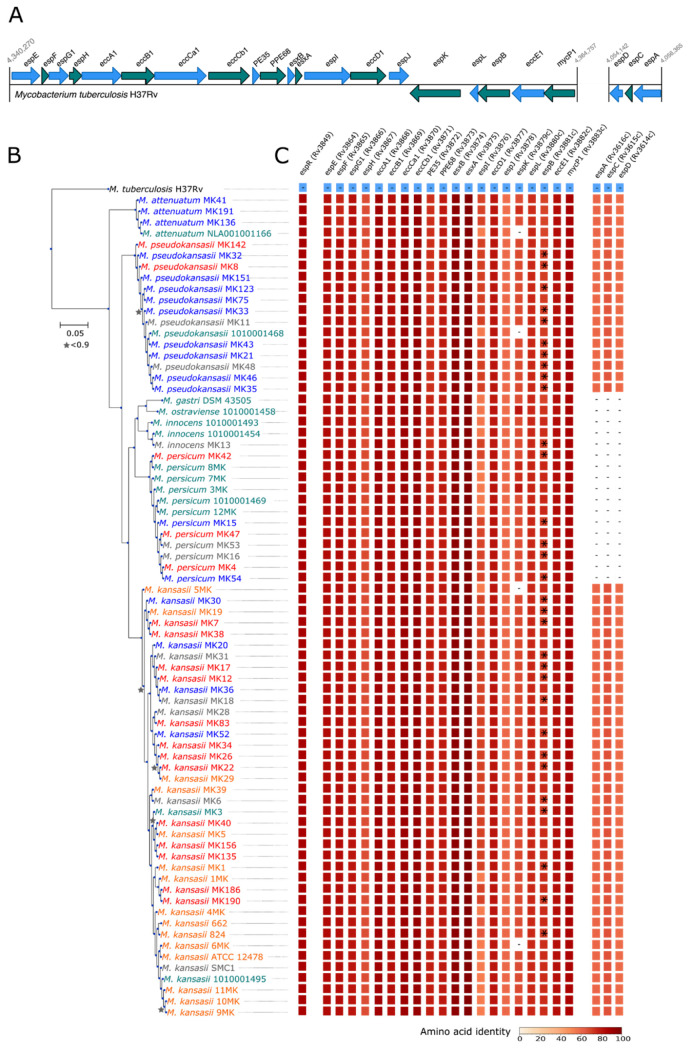
Maximum-likelihood core-genome phylogeny and conservation of the Type-VII secretion system-associated ESX-1 and *espABC* loci among members of the *M. kansasii* complex (**A**) ESX-1 and EspACD loci of *M. tuberculosis* H37Rv. Ecc, ESX-1 conserved components; Esp, ESX-1 secreted protein. EsxA, ESAT-6; EsxB, CFP-10. EspACD locus is required for the secretion and function of EsxA and EsxB. (**B**) Maximum-likelihood phylogeny based on the concatenated amino acid alignments of single-copy orthologous proteins forming the core genome. Node supports based on the Shimodaira-Hasegawa (SH) test are indicated with a star when below 0.9. The scale bar represents the number of amino acid substitutions per site alongside the branches. Species pathogenicity is color-coded according to the results reported in Table 1. Red = 1 = pathogenic, Orange = 2 = probably pathogenic, Grey = 3 = unknown, Green = 4 = probably non-pathogenic, Blue = 5 = non-pathogenic. (**C**) Conservation of the type-VII secretion system ESX-1 locus and EspACD in members of the *M. kansasii* complex. EspACD was absent from all isolates of *M. gastri*, *M. ostraviense*, *M. innocens* and *M. persicum*. The amino acid identity with the reference *M. tuberculosis* sequence (in blue) is indicated. Stars in the column of *espB* homologs indicate that the gene was split due to assembly gaps.

Genome sizes ranged from 6.03 to 6.9 Mb, which is 2 Mb larger than *M. tuberculosis* (Figure S1) and the GC content was comprised between 65.8% and 66.2%. The maximum-likelihood phylogeny, based on the concatenation of 2099 core orthogroup alignments (length of 729,256 amino acids), clustered each species of the *M. kansasii* complex into seven distinct clades (Figure 1B), consistent with a previous study [10]. *M. kansasii* and *M. innocens* isolates showed a greater genetic diversity than *M. persicum*, *M. pseudokansasii* or *M. attenuatum*. The heterogeneity of *M. kansasii* isolates may be due to the larger number of strains included as compared to other species and to particular local epidemiology, since three clades (MK30 to MK38, MK20 to MK18 and MK28 to MK29) contained only isolates from Lausanne. For *M. innocens*, the number of included strains is too small to be interpreted (*n* = 3).

### 3.3. Virulence Factors of the M. kansasii Complex

**Figure 2:**
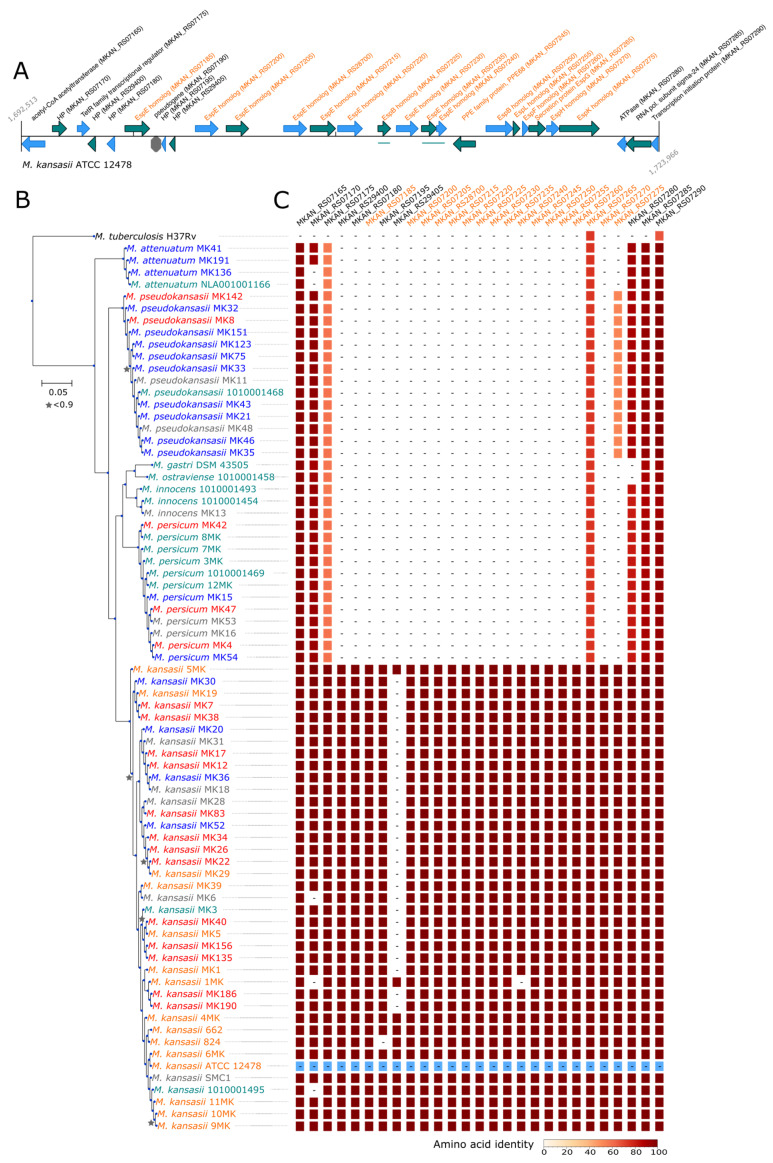
*M. kansasii*-specific genomic island encodes Type-VII secretion system effectors. (**A**) Locus is displayed for *M. kansasii* ATCC 12478, as a reference genome. Products mentioning “homolog” were manually annotated based on comparison to other genomes. Esp, ESX-1 secreted protein. All genes related to the type-VII secretion system are highlighted in orange. (**B**) Maximum-likelihood phylogeny based on the concatenated amino acid alignments of single-copy orthologous genes forming the core genome. Node supports based on the Shimodaira-Hasegawa (SH) test are indicated with a star when below 0.9. The scale bar represents the number of amino acid substitutions per site alongside the branches. Species pathogenicity is color-coded according to the results reported in Table 1. Red = 1 = pathogenic, Orange = 2 = probably pathogenic, Grey = 3 = unknown, Green = 4 = probably non-pathogenic, Blue = 5 = non-pathogenic. (**C**) The putative genomic island is specific to the *M. kansasii* species. The amino acid identity with the reference *M. kansasii* strain ATCC 12478 sequence (in blue) is color-coded.

The presence of homologs of known *M. tuberculosis* virulence factors of different categories was assessed: (1) ESX-1 locus (Figure 1A,C), (2) ESX-2/3/4/5 loci, (3) mycolic acid synthesis, (4) complex lipid synthesis, (5) other genes related to lipid synthesis, (6) catabolism of cholesterol, (7) cell envelope protein, mammalian cell entry operons, (8) lipoproteins, phagosomal arresting, (9) inhibition of apoptosis, (10) oxidative and nitrosative stress resistance, (11) proteases, (12) metal transporters, (13) PE/PPE family proteins, (14) two-component systems, (15) transcription factors and (16) sigma factors, as described in Forrellad et al. [56]. Most *M. tuberculosis* virulence factors were conserved in all genomes of the *M. kansasii* complex highlighting the close relatedness of these two clades (Data Sheet 1, *M. tuberculosis* virulence factors used as query to look for orthologous and paralogous genes among the *M. kansasii* complex). In addition, all strains encoded at least five distinct type-VII secretion systems (additional secretion system loci were found on plasmid, cf. below). Although *espB* has previously been described to be lacking in some isolates of the *M. kansasii* complex [13], this was probably due to assembly gaps, and the gene itself may still be functional. By contrast, the *espACD* operon was missing in *M. persicum*, *M. ostraviense*, *M. innocens* as well as in *M. gastri* (Figure 1C).

No orthogroup was found to be present only in pathogenic strains and absent from all non-pathogenic strains. Furthermore, no orthologous group was shared by all pathogenic strains and absent from all non-pathogenic strains of the individual species *M. kansasii*, *M. persicum* or *M. pseudokansasii*. Similarly, no orthologous group, InterPro domain or PFAM motif specific to *M. kansasii* strains was associated with tissue tropism, either for bone and joint infections or for urinary tract infections.

A total of 107 orthogroups were found to be specific and conserved in all *M. kansasii* strains. Forty-four of them only encoded hypothetical proteins, but many other groups could be related to virulence. Indeed, many genes were annotated as ESX-1 secretion-associated protein (Esp); EspE (10 orthologous groups), EspB (2 groups), EspF (1 group), EspL (1 group) and EspH (1 group). EspE and EspB homologs harbored WxG or LxxxD motifs known to be required for type-VII secretion (Figure S2) [62]. Both could be modelled using Phyre2 [63] and showed the typical helix-loop-helix structure of type-VII secretion effectors (Figure S2). In addition, four groups of PPE family proteins and one group of PE family proteins were likely substrates of the type-VII secretion system. Surprisingly, most of these homologs were located on a single genomic island present only in *M. kansasii* strains (Figure 2). In addition, this region encoded a homolog of EspG1, a chaperonin involved in the folding of effectors of the ESX-1 locus. This latter was not detected by the orthologous comparison because the orthologous group was shared by all strains; *M. kansasii* strains had two homologs, whereas other species of the complex only encoded one. Finally, one *espK* homolog, known to interact with EspB and EccCb1 (although not required for full virulence) [64], was also found in the mobile genetic element. Orthologs of EspK were also identified in *M. pseudokansasii* strains, but in a different genetic context, flanked with genes annotated as *espB*, *esxA*, *esxB* and *espK*.

### 3.4. Three Distributive Conjugal Transfers Induced Gene Losses Associated with Pathogenicity

To investigate the existence of genetic determinants supporting increased virulence in *M. kansasii* complex, a genome-wide association study was performed using treeWAS [53]. This tool takes into account the phylogeny to score associations between phenotypic data (e.g., virulence level) and genetic variations (e.g., SNPs or presence/absence of genes). Three scores are computed by treeWAS: the terminal, the simultaneous and the subsequent scores. The terminal score compares and correlates leaves phenotypes and genotypes, regardless of the ancestral information. The simultaneous score is based on the number of branches with parallel changes (phenotype and genotypes). Finally, the subsequent score computes the proportion of the tree with simultaneous changes in genotypes and phenotypes.

**Figure 3:**
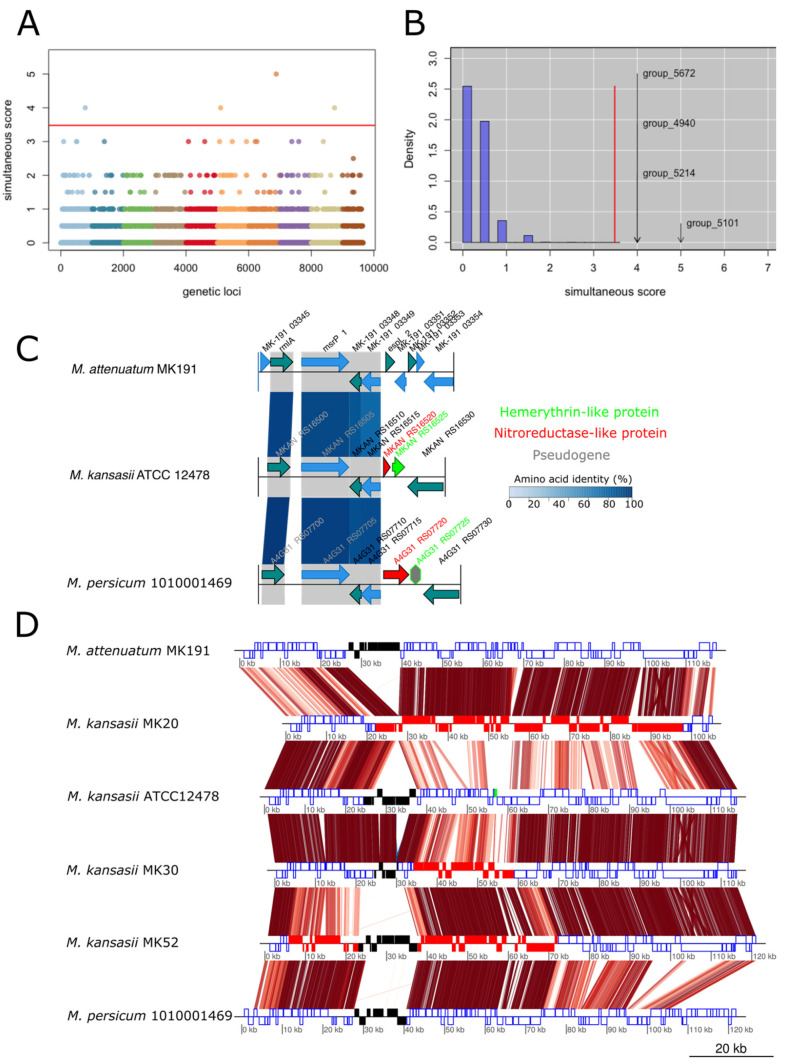
treeWAS results based on a matrix of presence/absence of single-copy orthologs. (**A**) Manhattan plot (colors were randomly assigned) and (**B**) bar plot of the treeWAS simultaneous score based on the presence/absence of single-copy orthologs and the core-genome phylogeny. Group_5101 and group_5214 encode for a hemerythrin-like protein and a nitroreductase-like protein, respectively, that were also identified by the terminal score. Group_5672 encodes a PE-family protein that is likely a substrate of the type-VII secretion system. Group_4940 encodes for a hypothetical protein that was negatively associated with pathogenicity. However, the gene is pseudogenized in some *M. kansasii* isolates and is thus most probably non-functional. (**C**) Genomic context of group_5101 and group_5214 in the representative genomes of *M. kansasii*, *M. persicum* and *M. attenuatum*. The hemerythrin-like protein was absent and pseudogenized in *M. attenuatum* and *M. persicum*, respectively. The nitroreductase-like protein was longer in *M. persicum* as compared to *M. kansasii* while it was absent in *M. attenuatum*. (**D**) The extended genomic region surrounding the hemerythrin-like protein (highlighted in green in strain ATCC 12478) present distributive conjugal transfers between *M. attenuatum* and *M. kansasii*, as well as between *M. persicum* and *M. kansasii*, leading to the loss of this protein in MK20, MK30 and MK52. Amino acid identity (from TBLASTX searches) between genomes is represented by the red stripes with a color gradient from 80% (light red) to 100% (dark red). ORFs corresponding to prophage sequences were manually highlighted based on gene annotation (not detected using PHASTER).

The association between the presence/absence of single-copy orthogroups with pathogenic (categories 1 and 2) and non-pathogenic (categories 4 and 5) phenotypes in the complete dataset was significant for four and two orthogroups (*p* < 0.00001) with the simultaneous score and the terminal score, respectively (Figure 3A,B). Two orthogroups (group_5101 and group_5214) were identified by both scores: a hemerythrin-like protein and a nitroreductase-like protein that could be involved in the resistance to oxidative stresses. Both proteins are encoded by neighboring genes that could form an operon in *M. kansasii* (Figure 3C). The third significant orthogroup (group_5672) encoded a PE family protein and is likely a substrate of the type-VII secretion system. The last orthogroup (group_4940) encoded a hypothetical protein that was negatively associated with pathogenicity. However, the gene was pseudogenized in *M. kansasii* isolates and should hence not be considered (Figure S3). When assessing the association of single nucleotide polymorphisms (SNPs) present in the core-genome of *M. kansasii* strains with the phenotype, no signal could be detected.

Three of the four non-pathogenic *M. kansasii* isolates (MK20, MK30 and MK52) lacked the orthogroups identified by GWAS analysis (Figure S3). The region surrounding the hemerythrin-like and the nitroreductase-like proteins showed evidence of distributive conjugal transfer (DCT) originating from *M. persicum* for MK30 (25 kb) and MK52 (67 kb) and from *M. attenuatum* for MK20 (75 kb) (Figure 3D). This indicates that distinct recombination events in an overlapping genomic region led to the loss of the hemerythrin-like and nitroreductase-like proteins. The hemerythrin-like protein was absent in *M. attenuatum* and truncated (A4G31_RS07725; 65 aa) by a frameshift mutation in *M. persicum* (Figure 3C). Another nitroreductase-like protein identified in *M. persicum* clustered in a different orthogroup and was longer than the *M. kansasii* homolog and absent in *M. attenuatum* (Figure 3C).

**Figure 4:**
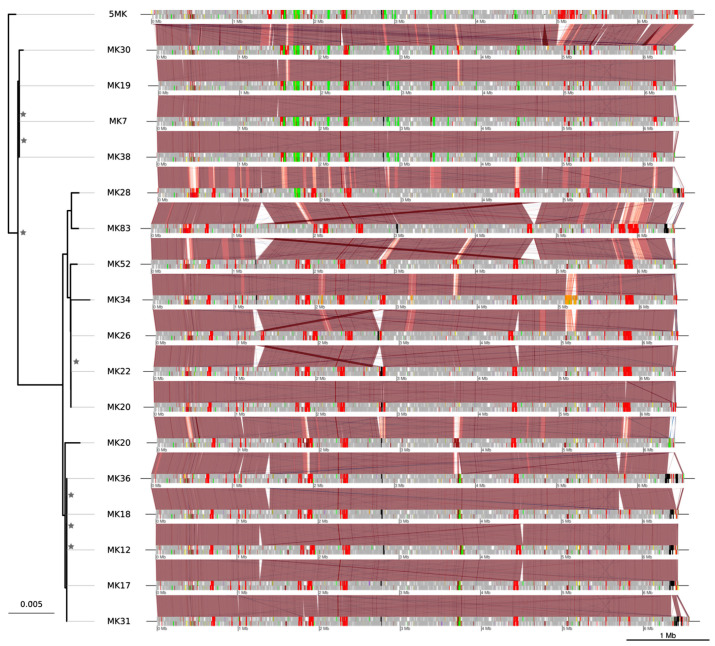
Distributive conjugal transfers within the species *M. kansasii.* For visual purposes, a subset of *M. kansasii* isolates was selected to illustrate the distributive conjugal transfers between members of the complex and the species *M. kansasii*. The remaining *M. kansasii* isolates are shown in Figure S4. The red stripes between genomes indicate aligned regions (BLASTN; *e*-value < 0.00001, nucleotide identity >87%), with a color gradient indicating the identity from 87% (light red) to 100% (dark red). Recombining loci are highlighted in bright red when the recombination occurred with *M. persicum*, in green with *M. pseudokansasii*, in orange with *M. ostraviense*, in yellow with *M. innocens*, in brown with *M. attenuatum* and in purple with *M. gastri*. Predicted prophages are highlighted in black. Some large DCTs between *M. persicum* or *M. pseudokansasii* and *M. kansasii* likely occurred in ancestral strains since they can be observed in the whole subset of strains (absent in Figure S4). Conversely, other large DCT, such as that between *M. innocens* and *M. kansasii* MK34, are likely more recent. Although all genomes displayed in this figure are draft genomes, they exhibit a high collinearity after contig reordering. The maximum-likelihood phylogeny of the strains is an extracted subset of the core-genome phylogeny shown in Figure 1B.

Since DCT likely explained variations in *M. kansasii* pathogenicity, we wanted to further assess the occurrence of distributive conjugal transfers (DCT) between all the members of the *M. kansasii* complex. For this purpose, coding sequences showing a higher average amino acid identity with another species than its own were considered to be probable recombinant regions, as highlighted in Figure 4 for a subset of *M. kansasii* isolates (see Figures S4–S7 for the remaining *M. kansasii* isolates, *M. persicum*, *M. pseudokansasii*, and *M. attenuatum*, respectively). DCT signals were widespread in the *M. kansasii* complex, involving recombination of one gene up to large genomic fragments. Overall, *M. kansasii* presented larger regions of DCTs as compared with *M. persicum*, *M. pseudokansasii* and *M. attenuatum*. Conversely, several *M. kansasii* isolates presented fewer DCT signals in a similar fashion to the other species of the complex (Figure S4). In some cases, the signal of DCT is conserved in entire clades, suggesting that some DCTs likely occurred in common ancestors of these clades, and were conserved throughout further individual evolution of the strains. On the contrary, some DCTs were restricted to one isolate, indicating more recent transfer events. Although they might be widespread, this method is not designed to allow the detection of transfers between strains of the same species. Furthermore, DCTs from species that are not included cannot be detected.

### 3.5. Conjugative Plasmids Are Widespread but Not Associated with Pathogenicity

**Figure 5:**
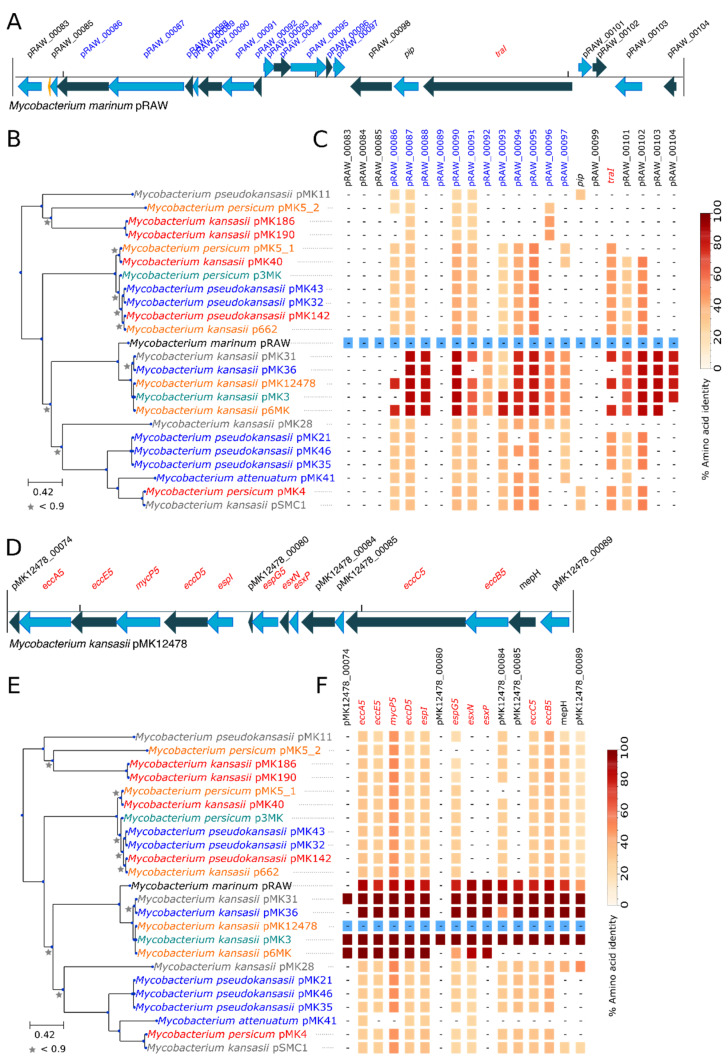
Distribution of the conjugative plasmids detected in the *M. kansasii* complex. (**A**) The type-IV secretion system locus (in blue), as well as the relaxase (*traI*) of the reference conjugative plasmid of *M. marinum* pRAW (as described in Ummels et al. [31]), is conserved (**C**) in some members of the *M. kansasii* complex. For multiple-copy orthologous groups, the amino acid identity of the best hit is shown. The reference sequences used to build this plot are indicated in blue. (**D**) Schematic representation of the plasmid-encoded Type-VII secretion system of strain ATCC 12478 and its gene conservation (**F**) in members of the *M. kansasii* complex. (**B**,**E**) Maximum-likelihood phylogeny based on the concatenated alignment of six single-copy orthologous genes (2766 amino acids) of the plasmid dataset. Bootstrap values below 0.9 are indicated with grey stars. The colors of the labels indicate pathogenicity, as reported in Table 1. Red = 1 = pathogenic, Orange = 2 = probably pathogenic, Grey = 3 = unknown, Green = 4 = probably non-pathogenic, Blue = 5 = non-pathogenic. Closely related plasmids sharing >99% of amino acid identities were found in different species, indicating probable recent conjugative transfers between species of the complex.

Conjugative plasmids were found in 22 out of the 75 strains of the *M. kansasii* complex (29.3%). Two conjugative plasmids were complete and circularized publicly available sequences (pMK12478 and pMK142). Interestingly, strain MK5 presented two different plasmids. All detected plasmids shared the same backbone structure as described by Ummels et al., although the approach used to identify plasmids likely biased their selection [31]. Only four plasmids lacked the *traA/traI* homolog but still shared type-VII and type-IV secretion systems homologs (Figure 5). As indicated by the high level of conservation (>99% of amino acid identities) between several plasmids as well as by the plasmid phylogeny, relatively recent conjugative plasmid transfers occurred between different isolates and different species of the *M. kansasii* complex (Figures S9 and S10). Sizes ranged from 60 Kbp to 170 Kbp. The plasmid sizes may have been underestimated because some contigs could have been missed during the inclusion process despite the use of various backbone genes and GC-coverage plots. The mean GC content of plasmids was significantly lower than that of chromosomes (*p*-value < 0.00001, two sample *t*-test) (Figure S8).

The presence of plasmids was not associated with variations in pathogenicity (*p*-value = 0.91, Chi-square). Furthermore, the presence/absence of single-copy orthogroups was assessed previously with the treeWAS analysis and returned no plasmid-encoded hit. Conjugative plasmids exhibited various gene functions within and between plasmids as shown by the diversity of COG categories found (Figure S11). Interestingly, a type I-E CRISPR-*cas* system locus was found on the plasmid of *M. pseudokansasii* MK11 (Figure S12). Surprisingly, the best BLAST hits (using BLASTP) with the non-redundant protein database of the NCBI were found in the distantly related *M. abscessus* species. Such CRISPR-*cas* loci can be found on mobile genetic elements [65], but the transfer between a fast and a slow growing mycobacterium was unexpected.

## 4. Discussion

The *M. kansasii* complex currently regroups seven closely related species sharing nearly 90% average nucleotide identity. Their prevalence and pathogenicity vary significantly [11,66,67,68,69], but as observed in our dataset as well, *M. kansasii* is the most frequently isolated and most pathogenic species. The unexpected isolation of two *M. pseudokansasii* strains in urine specimens could suggest a particular tropism in this species, which remains to be confirmed in larger studies. The comparative genomic analysis revealed several elements that may contribute to the differences in pathogenicity between members of the *M. kansasii* complex. First, *M. persicum*, *M. ostraviense*, *M. innocens* and *M. gastri* lacked the operon encoding for EspACD, which are major virulence factors of *M. tuberculosis* [70]. Indeed, in *M. tuberculosis*, the secretion of ESAT-6 and CFP-10 effectors requires the expression of EspA [19,70] and maintenance of wild-type levels of EspA require both EspC and EspD [71]. Recently, it has even been postulated that EspC could act as the needle of the type-VII secretion system apparatus [72]. Thus, in light of the conservation of other known virulence factors, the absence of the *espACD* operon could have a role in the reduced pathogenicity of these species and in their rare isolation from patients. However, both *M. pseudokansasii* and *M. attenuatum—*two mostly non-pathogenic species—also encoded this locus, and hence additional genetic factors may explain the increased virulence of *M. kansasii*.

Going along with this hypothesis, *M. kansasii* harbored a large genomic island, encoding 17 effectors of the type-VII secretion system that could play a role in the virulence and the interaction of *M. kansasii* strains with its host. Although *M. pseudokansasii* exhibited another genomic region encoding putative type-VII secretion system effectors, it was much shorter (1 *espK*, 1 *espB* and 2 WxG-100-familly proteins homologs). Further studies using mutant and wild type strains will be needed to investigate the role of these genetic factors in the pathogenicity of the *M. kansasii* complex.

In *M. tuberculosis*, variation in virulence were seen after functional modifications of master regulators of virulence, such as the PhoPR or the DosR regulon [56,73]. However, all regulators investigated (Data sheet 1) shared the same amino acid identities within species and no single nucleotide polymorphism (SNPs) of the *M. kansasii* species was significantly associated with pathogenicity. However, two genes, encoding hemerythrin-like and nitroreductase-like proteins, were significantly associated with pathogenic phenotypes (Figure 3). In *M. tuberculosis*, the hemerythrin-like protein Rv2633c is one of the genes that are up-regulated upon infection into THP-1 macrophages [74,75]. Surprisingly, Rv2633c was recently shown not to bind O_2_, as could have been expected from a hemerythrin, but exhibited a catalase activity [76]. Although the *M. kansasii* hemerythrin-like protein did not cluster with the same orthogroup as Rv2633c, it could play a similar role as it is located upstream a nitroreductase-like protein, and genes encoded in operon frequently harbor related functions [77]. Since protection against reactive oxygen and reactive nitrogen species are critical for the intracellular survival in macrophages, we hypothesize that the loss of these genes could cause a decreased virulence.

Among possible evolutionary processes that could lead to hemerythrin and nitroreductase gene loss, DCTs represent the most likely mechanism for the following reasons. The large region affected by transfer precludes classical transformation mechanisms that usually involve the transfer of single small DNA fragments (3–5 Kb) [28]. Indeed, DCT can mobilize several DNA fragments of up to 200 Kb, creating mosaic genomes through recombination [28,35]. *M. kansasii* strains MK30 and MK52 likely received multiple DCTs from *M. persicum* (in red in Figure 4) whereas *M. kansasii* MK20 harbored several large recombining regions from *M. attenuatum* (in brown in Figure 4). Moreover, these regions were not prophages based on a manual inspection of the annotation and on PHASTER predictions, nor integrated chromosomal conjugative plasmids (episomes), ruling out transduction and classical conjugation, respectively.

The similarity of conjugative plasmids across different species of the *M. kansasii* complex suggests the occurrence of HGTs most likely through conjugation among and within species. This is corroborated by the phylogenetic reconstruction based on core-plasmid genes that intertwines plasmids belonging to different species in the same clades. In the present dataset, the pathogenicity of the strains could not be correlated with the presence or absence of plasmids, or some of their genetic content, but we cannot exclude an eventual role in bacterial pathogenicity. Indeed, the incomplete nature of genome assemblies often renders difficult the identification of all contigs forming the plasmids, and the method used here could have biased our analysis towards the identification of previously known conjugative plasmids.

Overall, comparative analyses and genome-wide association studies on 74 genomes could identify several loci likely associated with the increased or decreased pathogenicity in species of the *M. kansasii* complex, as well as among *M. kansasii* strains. DCTs, conjugative plasmid transfers and transduction were frequently observed and several evidence indicate that DCT was directly involved in gene losses affecting strain pathogenicity. While DCT was thought to be an important mechanism for HGT in mycobacteria [28], it has, to our knowledge, never previously been directly associated with a decreased pathogenicity. This demonstrates the previously unsuspected importance of HGT between and within species, conferring a high level of genome plasticity to these non-tuberculous mycobacteria, and likely providing a selective advantage and faster genome evolution. The existence and the extent of such genetic transfers with other mycobacteria remain to be investigated by including further distantly related species in similar comparative analyses.

## Figures and Tables

**Table 1:** Included strains of the *M. kansasii* complex and pathogenicity.

Strain ID	Spec.	Age	Diagnosis	Co-Morbidities	P.	I.	O.	Species	Or.
1010001468	Environm.	NA	NA	NA	4	NA	0	*M. attenuatum*	0
MK136	ND	ND	Colonization	ND	5	ND	0	*M. attenuatum*	1
MK191	ND	ND	Colonization	ND	5	ND	0	*M. attenuatum*	1
MK41	Bronch. asp.	48	Colonization	Lung transplanted	5	1	0	*M. attenuatum*	2
NLA001001166	ND	ND	Colonization	Bronchectasis	4	ND	0	*M. attenuatum*	0
1010001458	Environm.	NA	NA	NA	4	NA	0	*M. ostraviense*	0
DSM43505	Gastric lavage	ND	Colonization	ND	4	ND	0	*M. gastri*	0
1010001454	Environm.	NA	NA	NA	4	NA	0	*M. innocens*	0
1010001493	Environm.	NA	NA	NA	4	NA	0	*M. innocens*	0
MK13	Sputum	87	ND	ND	3	ND	0	*M. innocens*	2
662	BAL/Bronch. asp.	ND	Lung NTM disease	ND	2	ND	0	*M. kansasii*	0
824	Sputum	ND	Lung NTM disease	ND	2	ND	0	*M. kansasii*	0
1010001495	Environm.	NA	NA	NA	4	NA	0	*M. kansasii*	0
10 MK	Sputum	ND	Lung NTM disease	ND	2	ND	0	*M. kansasii*	0
11 MK	BAL	ND	Lung NTM disease	ND	2	ND	0	*M. kansasii*	0
1 MK	Sputum	ND	Lung NTM disease	ND	2	ND	0	*M. kansasii*	0
4MK	Sputum	ND	Lung NTM disease	ND	2	ND	0	*M. kansasii*	0
5MK	BAL	ND	Lung NTM disease	ND	2	ND	0	*M. kansasii*	0
6MK	Sputum	ND	Lung NTM disease	ND	2	ND	0	*M. kansasii*	0
9MK	BAL	ND	Lung NTM disease	ND	2	ND	0	*M. kansasii*	0
ATCC12478	ND	ND	NTM disease	ND	2	ND	0	*M. kansasii*	0
MK1	BAL	50	Lung NTM disease	ND	2	ND	0	*M. kansasii*	2
MK12	Sputum	19	Lung NTM disease	-	1	1	0	*M. kansasii*	2
MK135	ND	ND	Lung NTM disease	ND	1	ND	0	*M. kansasii*	1
MK156	ND	ND	Lung NTM disease	ND	1	ND	0	*M. kansasii*	1
MK17	Bronch. asp.	71	Lung NTM disease	Lung adenocarcinoma, bronchectasis	1	0	0	*M. kansasii*	2
MK18	Bronch. asp.	67	ND	ND	3	ND	0	*M. kansasii*	2
MK186	ND	ND	Lung NTM disease	ND	1	ND	0	*M. kansasii*	1
MK19	Sputum	68	Lung NTM disease	ND	2	ND	0	*M. kansasii*	2
MK190	ND	ND	Lung NTM disease	ND	1	ND	0	*M. kansasii*	1
MK20	Sputum	16	Colonization	Cystic fibrosis	5	0	0	*M. kansasii*	2
MK22	Biopsy	27	Tenosynovitis, flexor of the 3rd finger	History of toxic pneumonitis due to hydrogen chroride exposition	1	0	1	*M. kansasii*	2
MK26	Biopsy	23	Finger infection (no further information)	ND	1	ND	1	*M. kansasii*	2
MK28	Bronch. asp.	64	ND	ND	3	ND	0	*M. kansasii*	2
MK29	BAL	58	ND	ND	2	ND	0	*M. kansasii*	2
MK3	BAL	77	ND	Lung adenocarcinoma	4	0	0	*M. kansasii*	2
MK30	Bronch. asp.	67	Colonization	Lung adenocarcinoma, COPD, liver cirrhosis, valvular and rythmic cardiopathy	5	0	0	*M. kansasii*	2
MK31	Bronch. asp.	63	ND	Rhumatoid arthritis	3	1	0	*M. kansasii*	2
MK34	Sputum	82	Lung NTM disease	Rhumatoid arthritis with lung involvement	1	1	0	*M. kansasii*	2
MK36	Sputum	59	Colonization	Primary ciliary dyskinesia, bronchectasis	5	0	0	*M. kansasii*	2
MK38	Surgical spec.	47	Sternitis	Rhumatoid arthritis	1	1	1	*M. kansasii*	2
MK39	Sputum	49	Lung NTM disease	ND	2	ND	0	*M. kansasii*	2
MK40	Surgical spec.	49	Hand infection (no further information)	Chronic hip prosthesis infection with *Propionibacterium acnes*	1	0	1	*M. kansasii*	2
MK5	Sputum	32	Lung NTM disease	ND	2	ND	0	*M. kansasii*	2
MK52	Sputum	66	Colonization	Lung adenocarcinoma, COPD	5	0	0	*M. kansasii*	2
MK6	Sputum	73	ND	ND	3	ND	0	*M. kansasii*	2
MK7	Sputum	34	Lung NTM disease	Chronic hepatitis B treated with alfa-2a peginterferon	1	1	0	*M. kansasii*	2
MK83	ND	ND	Lung NTM disease	ND	1	ND	0	*M. kansasii*	1
SMC1	ND	ND	Human-environ.	ND	3	ND	0	*M. kansasii*	0
1010001469	Environm.	NA	NA	NA	4	NA	0	*M. persicum*	0
12MK	BAL	ND	Colonization	ND	4	ND	0	*M. persicum*	0
3MK	BAL	ND	Colonization	ND	4	ND	0	*M. persicum*	0
7MK	Sputum	ND	Colonization	ND	4	ND	0	*M. persicum*	0
8MK	BAL	ND	Colonization	ND	4	ND	0	*M. persicum*	0
MK15	Endotracheal secretions	8	Colonization	Still’s disease	5	1	0	*M. persicum*	2
MK16	Sputum	29	Colonization	Disseminated *M. avium* infection	3	1	0	*M. persicum*	2
MK4	Bronch. asp.	28	NTM disease	Acute lymphoblastic leukemia, bone marrow allografted	1	1	0	*M. persicum*	2
MK42	Surgical spec.	32	Chronic olecranon bursitis	ND	1	ND	1	*M. persicum*	2
MK47	Bronch. asp.	77	Lung NTM disease	Polymyalgia rhumatica	1	1	0	*M. persicum*	2
MK53	Sputum	76	ND	ND	3	ND	0	*M. persicum*	2
MK54	Bronch. asp.	27	Colonization	Bronchectasis	5	0	0	*M. persicum*	2
MK11	Urine	27	Colonization	Recurent UTI	3	0	0	*M. pseudokansasii*	2
MK123	ND	ND	Colonization	ND	5	ND	0	*M. pseudokansasii*	1
MK142	Blood culture	ND	Bacteremia, disseminated disease	ND	1	ND	0	*M. pseudokansasii*	1
MK151	ND	ND	Colonization	ND	5	ND	0	*M. pseudokansasii*	1
MK21	Sputum	52	Colonization	History of community-acquired pneumococcal pneumonia	5	0	0	*M. pseudokansasii*	2
MK32	Sputum	80	Colonization	Small cell lung carcinoma, history of radiation pneumonitis and *M. tuberculosis* infection	5	0	0	*M. pseudokansasii*	2
MK33	Bronch. asp.	60	Colonization	Small cell lung carcinoma	5	0	0	*M. pseudokansasii*	2
MK35	Sputum	47	Colonization	History of *M. tuberculosis* infection, Barret’s oesophagus, rectal adenocarcinoma	5	0	0	*M. pseudokansasii*	2
MK43	Bronch. asp.	9	Colonization	Miller-Dieker syndrome	5	0	0	*M. pseudokansasii*	2
MK46	Sputum	30	Colonization	History of *M. tuberculosis* infecction	5	0	0	*M. pseudokansasii*	2
MK48	Sputum	33	ND	ND	3	ND	0	*M. pseudokansasii*	2
MK75	ND	ND	Colonization	ND	5	ND	0	*M. pseudokansasii*	1
MK8	Urine	51	UTI	Renal transplanted	1	1	0	*M. pseudokansasii*	2

Strain ID, Strain identifier; Spec., specimen; ND, not determined; NA, not attributable; UTI, Urinary tract infection; BAL, Bronchoalveolar lavage; Bonch. asp., bronchial aspiration; Environm. Environmental; Diagnosis, diagnosis related to the isolated *M. kansasii* complex species; NTM, Non-tuberculosis mycobacteria; P., Pathogenicity; 1 = pathogenic, 2 = probably pathogenic, 3 = unknown, 4 = probably non-pathogenic/colonizer, 5 = non-pathogenic/colonizer.; I. Immunosuppression, 1 = yes, 0 = no; O., Osteoarticular presentation, 1 = yes, 0 = no; Or., origin (source), 0 = publicly available genome from the NCBI, 1 = strain used by Goy et al., 2 = this study.

## Data Availability

All sequencing data are available under the BioProject accession numbers PRJEB25525 and PRJEB28857.

## Supplementary Materials

The following are available online at https://www.mdpi.com/2076-2607/9/2/348/s1, Figure S1: Core-genome phylogeny and genomic features; Figure S2: *espE* homologs of *M. kansasii* located on the genomic island described in Figure 2; Figure S3: Distribution of the homologs of the best treeWAS hits; Figure S4: Distributive conjugal transfer events among the *M. kansasii* species; Figure S5: Distributive conjugal transfer events among the *M. persicum* species; Figure S6: Distributive conjugal transfer events among the *M. pseudokansasii* species; Figure S7: Distributive conjugal transfer events among the *M. attenuatum* species; Figure S8: Genomic features of the conjugative plasmids; Figure S9: Alignment of the complete plasmid sequence of *M. kansasii* strain ATCC 12478 and four closely related plasmids of the dataset; Figure S10: Alignment of the complete plasmid sequence of *M. pseudokansasii* strain MK142 and six closely related plasmids of the dataset; Figure S11: Cluster of Orthologous Genes (COG) annotated on the identified conjugative plasmids; Figure S12: Plasmid-encoded CRISPR-cas system; Data sheet 1: *M. tuberculosis* virulence factors used as query to look for orthologous and paralogous genes among the *M. kansasii* complex [78,79,80].
