# Supplementary material for: Pathogenic Determinants of the Mycobacterium kansasii Complex: An Unsuspected Role for Distributive Conjugal Transfer
Source: Microorganisms. 2021 Feb 10;9(2):348. doi: 10.3390/microorganisms9020348 (PMC7916490; doi:10.3390/microorganisms9020348)
Supplement: Supplementary file 1 [file microorganisms-09-00348-s001.zip › Supplementary_materials/Supplementary_materials.pdf]

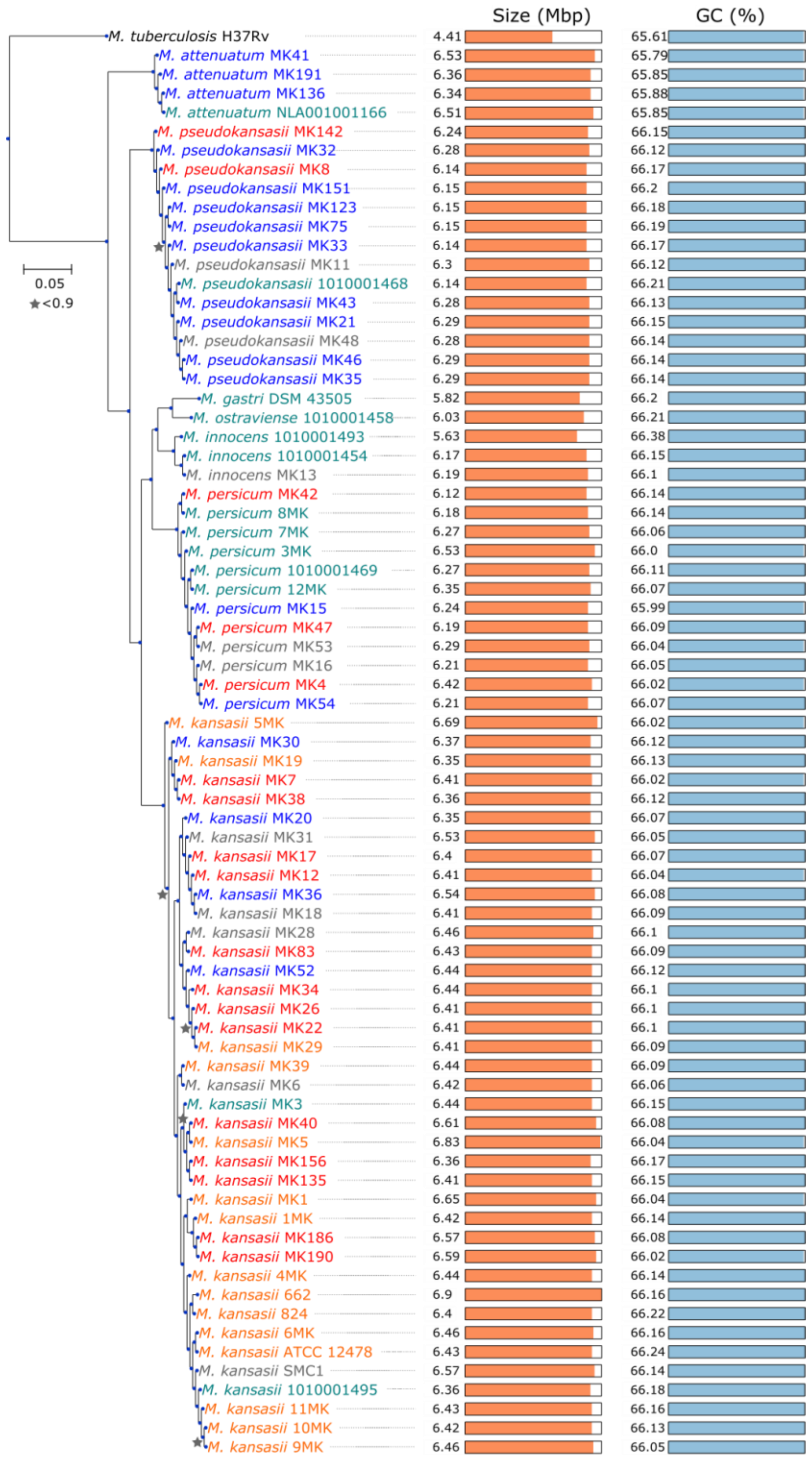

Figure S1 – core-genome phylogeny and genomic features

Bootstrap values below 0.9 are indicated with a star. The root of the tree was determined using *M. tuberculosis* H37Rv as a reference genome. *M. ostraviense*, *M. innocens* and *M. gastri* tended to exhibit shorter genomes but no conclusion should be made (draft genomes and small sample size). The GC content was similar between all members of the *M. kansasii* complex.

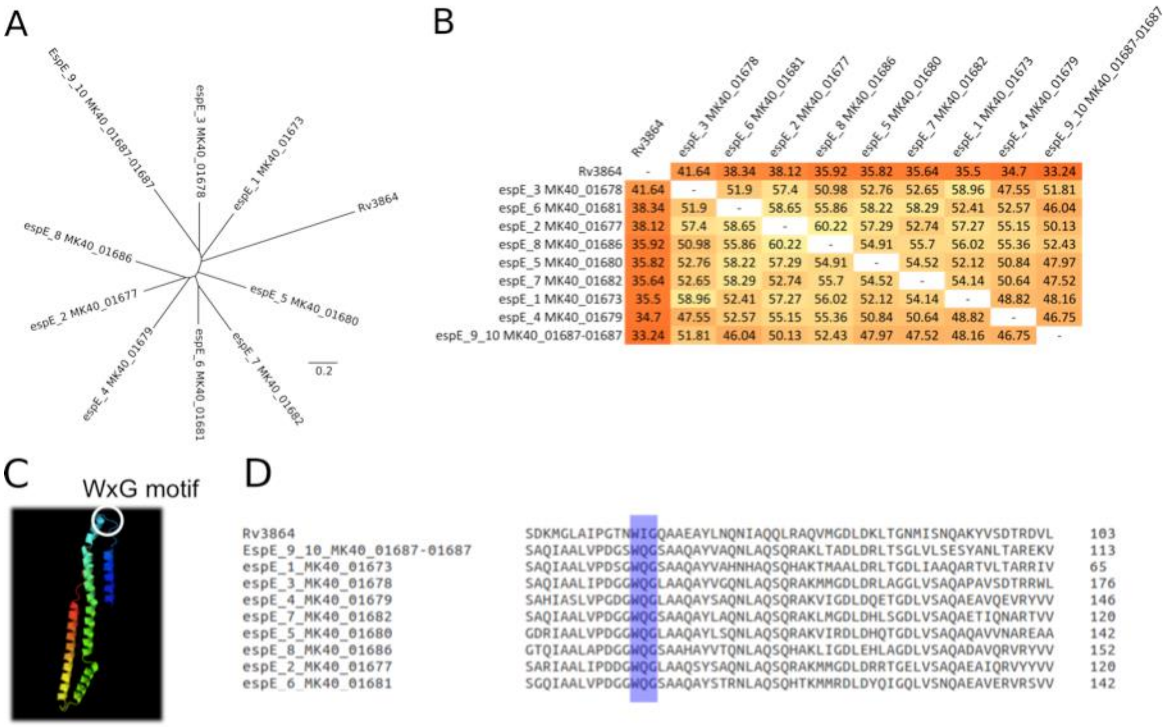

Figure S2 – *espE* homologs of *M. kansasii* located on the genomic island described in Figure 2.

A: Here is shown a maximum-likelihood phylogeny of the espE homologs of the genomic island described in Figure 2. *espE_9* and *espE_10* are split genes and were fused to be aligned with the rest of the espE genes using ClustalO **[78]** The phylogeny was reconstructed using FastTree v2.1.8 with standard parameters. B: The matrix of amino acid identities is based on the ClustalO alignment. C: The tertiary protein structure of EspE_1 (MK40_01673) was modelled using Phyre2 **[63]** and presents the classical helix-turn-helix of type-VII secretion system effectors. D: Part of the ClustalO alignment showing the classical WxG motif (highlighted in blue) found in type-VII secretion system effectors.

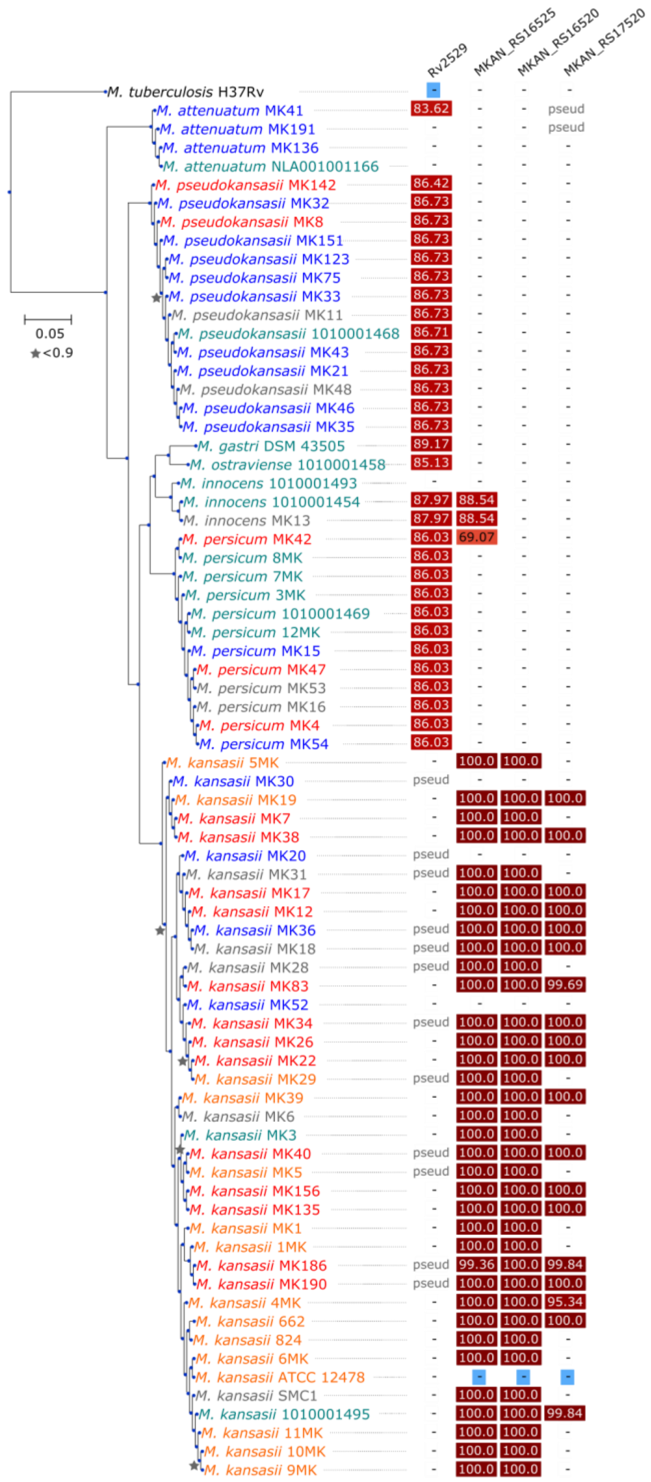

Figure S3 – Distribution of the homologs of the best treeWAS hits.

Distribution of the single-copy orthologous groups detected with treeWAS. The blue squares indicate the reference sequence to calculate the amino acid identity. The maximum-likelihood core-genome phylogeny is

the same as the one displayed in Figure 1B. Red=1=pathogenic, Orange=2=probably pathogenic, Grey=3=unknown, Green=4=probably non-pathogenic, Blue=5=non-pathogenic. Rv2529 encodes for a hypothetical protein and was detected as negatively associated with pathogenicity. However, the gene was a false positive hit because it was considered present in some *M. kansasii* isolates while it was in fact pseudogenized. MKAN_RS16525 and MKAN_RS16520 encode for the hemerythrin-like protein and the putative NADPH reductase shown in Figure 3C. Finally, MKAN_RS17520 encodes for a PE family protein and is likely a substrate of the type-VII secretion system.

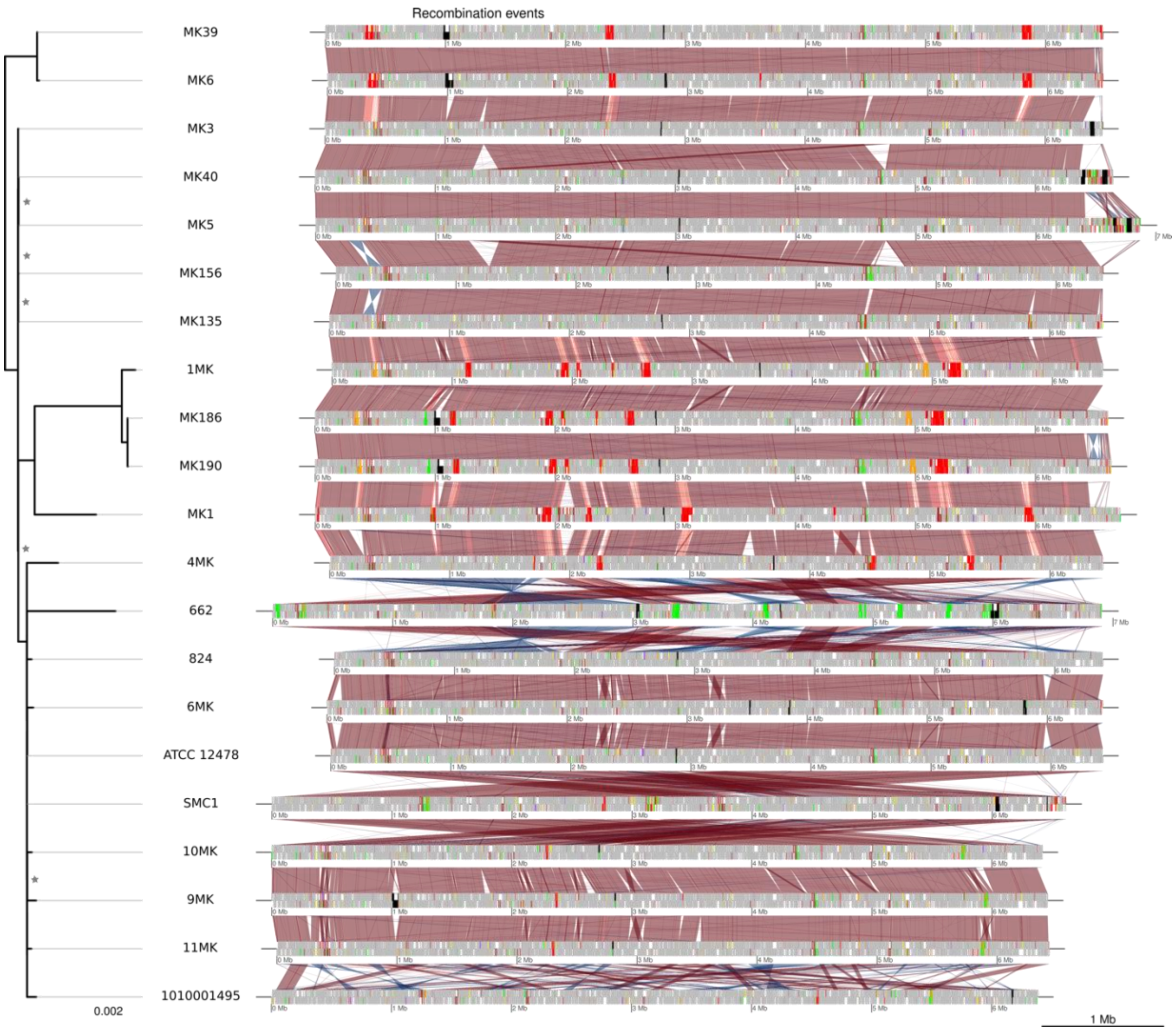

Figure S4 – Distributive conjugal transfer events among the *M. kansasii* species

Here is shown the second subset of the *M. kansasii* isolates and the detected distributive conjugal transfers (DCT) between the members of the complex and the species *M. kansasii*. The rest of the *M. kansasii* isolates are shown in Figure 4. The nucleotide sequence of each genome was aligned using BLASTN (e-value cut-off 0.00001, nucleotide identity cut-off 87%). The red stripes between genomes indicate the aligned regions, whereas the colour gradient indicates the identity from 87% (light red) to 100% (dark red). Recombining loci are highlighted in bright red when the recombination occurred with *M. persicum*, in green with *M. pseudokansasii*, in orange with *M. ostraviense*, in yellow with *M. innocens*, in brown with *M. attenuatum* and in purple with *M. gastri*. Regions of predicted prophages are highlighted in black. For some isolates, such as MK3, MK40 and MK5, less recombining loci were detected. Conversely, other large DCTs, such as the one between *M. pseudokansasii* and *M. kansasii* 662, were extensive. Two publicly available genomes did not show collinearity because contigs were not reordered. The maximum-likelihood phylogeny of the strains is an extracted subset of the core-genome phylogeny shown in Fig 1B.

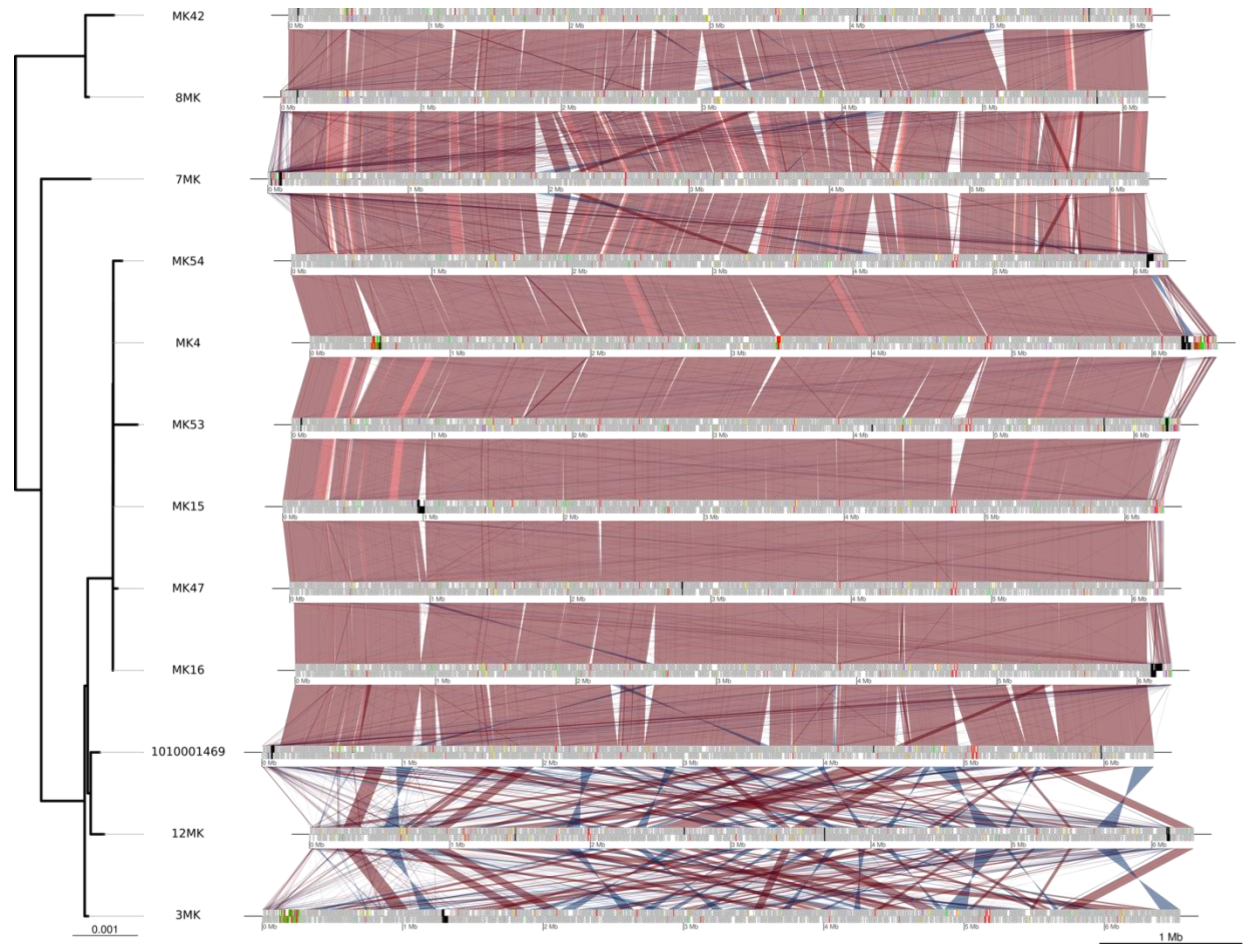

Figure S5 – Distributive conjugal transfer events among the *M. persicum* species

Here is shown the detected distributive conjugal transfers (DCT) between the members of the complex and the species *M. persicum*. The nucleotide sequence of each genome was aligned using BLASTN (e-value cut-off 0.00001, nucleotide identity cut-off 95%). The red stripes between genomes indicate the aligned regions, whereas the colour gradient indicates the identity from 95% (light red) to 100% (dark red). Recombining loci are highlighted in bright red when the recombination occurred with *M. kansasii*, in green with *M. pseudokansasii*, in orange with *M. ostraviense*, in yellow with *M. innocens*, in brown with *M. attenuatum* and in purple with *M. gastri*. Regions of predicted prophages are highlighted in black. Unlike *M. kansasii*, *M. persicum* exhibited less DCT using our dataset. Interestingly, DCTs may have occurred between a bacterium that was not present in the dataset and M. *persicum* strains MK53, MK54 and 7MK because large regions shared less nucleotide identity without being tagged as recombining regions. Two publicly available genomes did not show collinearity because contigs were not reordered. The maximum-likelihood phylogeny of the strains is an extracted subset of the core-genome phylogeny shown in Fig 1B.

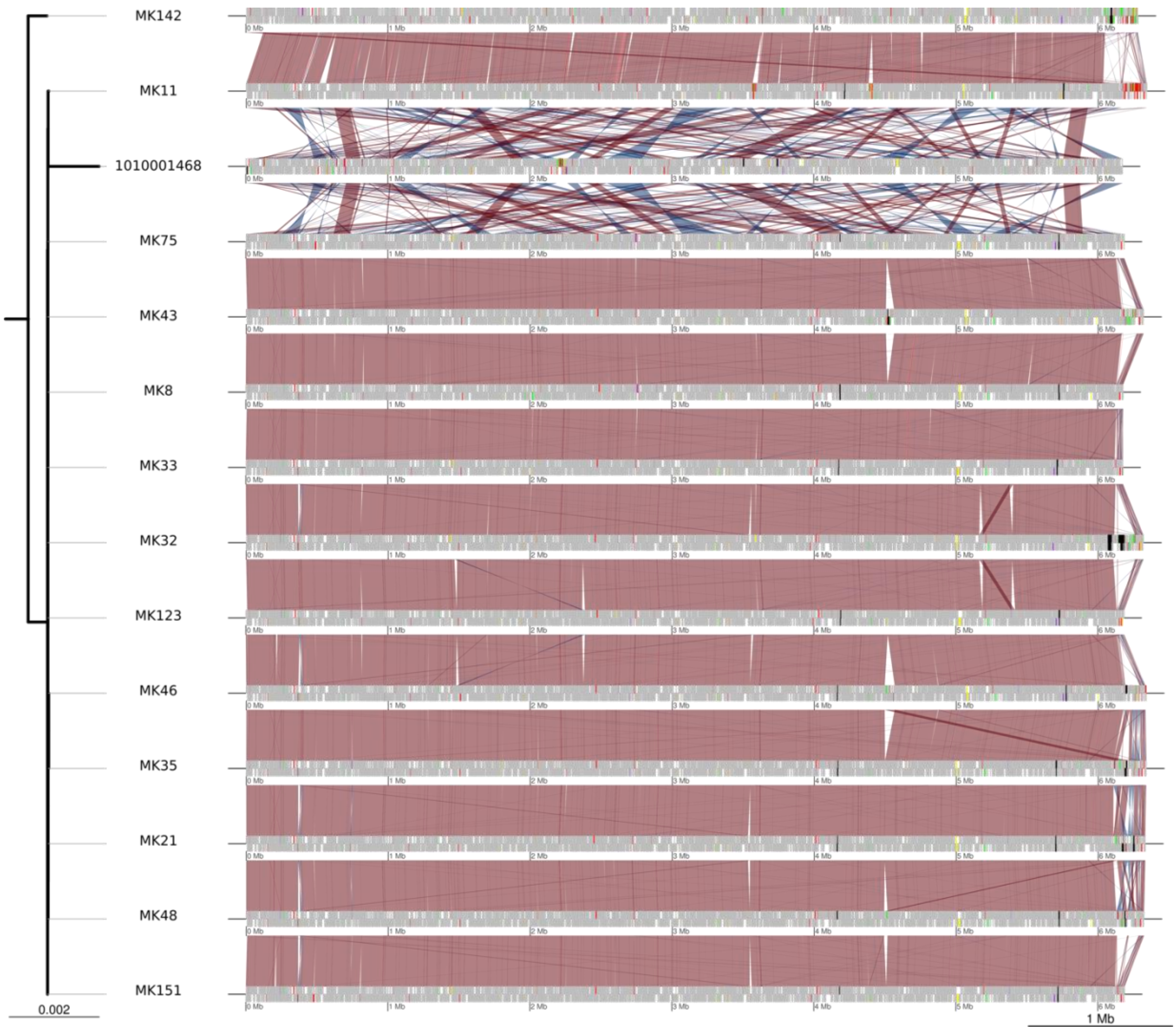

Figure S6 – Distributive conjugal transfer events among the *M. pseudokansasii* species

Here is shown the detected distributive conjugal transfers (DCT) between the members of the complex and the species *M. pseudokansasii*. The nucleotide sequence of each genome was aligned using BLASTN (e-value cut-off 0.00001, nucleotide identity cut-off 95%). The red stripes between genomes indicate the aligned regions, whereas the colour gradient indicates the identity from 95% (light red) to 100% (dark red). Recombining loci are highlighted in bright red when the recombination occurred with *M. kansasii*, in green with *M. persicum*, in orange with *M. ostraviense*, in yellow with *M. innocens*, in brown with *M. attenuatum* and in purple with *M. gastri*. Regions of predicted prophages are highlighted in black. Unlike *M. kansasii* and as *M. persicum*, *M. pseudokansasii* exhibited less DCT using our dataset. One publicly available genome did not show collinearity because contigs were not reordered. The maximum-likelihood phylogeny of the strains is an extracted subset of the core-genome phylogeny shown in Fig 1B.

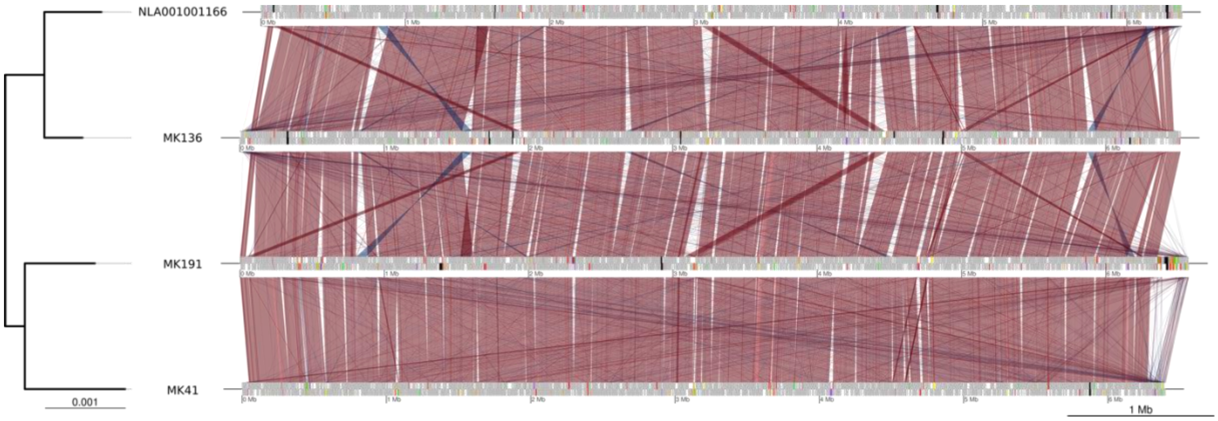

Figure S7 – Distributive conjugal transfer events among the *M. attenuatum* species

Here is shown the detected distributive conjugal transfers (DCT) between the members of the complex and the species *M. attenuatum*. The nucleotide sequence of each genome was aligned using BLASTN (e-value cut-off 0.00001, nucleotide identity cut-off 95%). The red stripes between genomes indicate the aligned regions, whereas the colour gradient indicates the identity from 95% (light red) to 100% (dark red). Recombining loci are highlighted in bright red when the recombination occurred with *M. kansasii*, in green with *M. persicum*, in orange with *M. pseudokansasii*, in yellow with *M. ostraviense*, in brown with *M. innocens* and in purple with *M. gastri*. Regions of predicted prophages are highlighted in black. Although all genome displayed in this figure are draft genomes, they exhibit a high collinearity after the contig reordering. The maximum-likelihood phylogeny of the strains is an extracted subset of the core-genome phylogeny shown in Fig 1B.

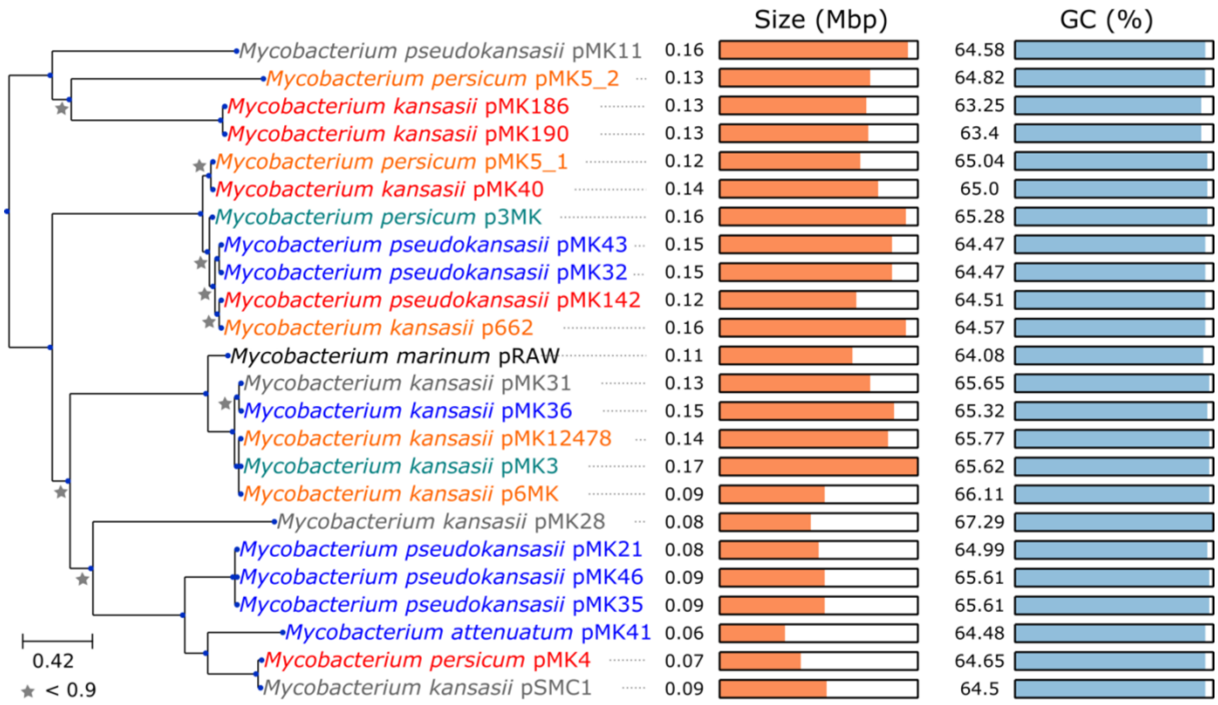

Figure S8 – Genomic features of the conjugative plasmids.

A maximum likelihood based on the concatenated alignment of single-copy orthologous genes of the plasmid dataset is shown. Bootstrap values below 0.9 are indicated with grey stars. A maximum likelihood phylogeny based on the concatenated alignment of single-copy orthologous genes (2,766 amino acids) of the plasmid dataset is shown. Bootstrap values below 0.9 are indicated with grey stars. Colours of the labels indicate pathogenicity as reported in Table 1. Red=1=pathogenic, Orange=2=probably pathogenic, Grey=3=unknown, Green=4=probably non-pathogenic, Blue=5=non-pathogenic. Genome sizes and GC contents are shown on the right panel. GC contents of the conjugative plasmids were significantly lower than the chromosomal ones ($p < 0.00001$, two sample t-test).

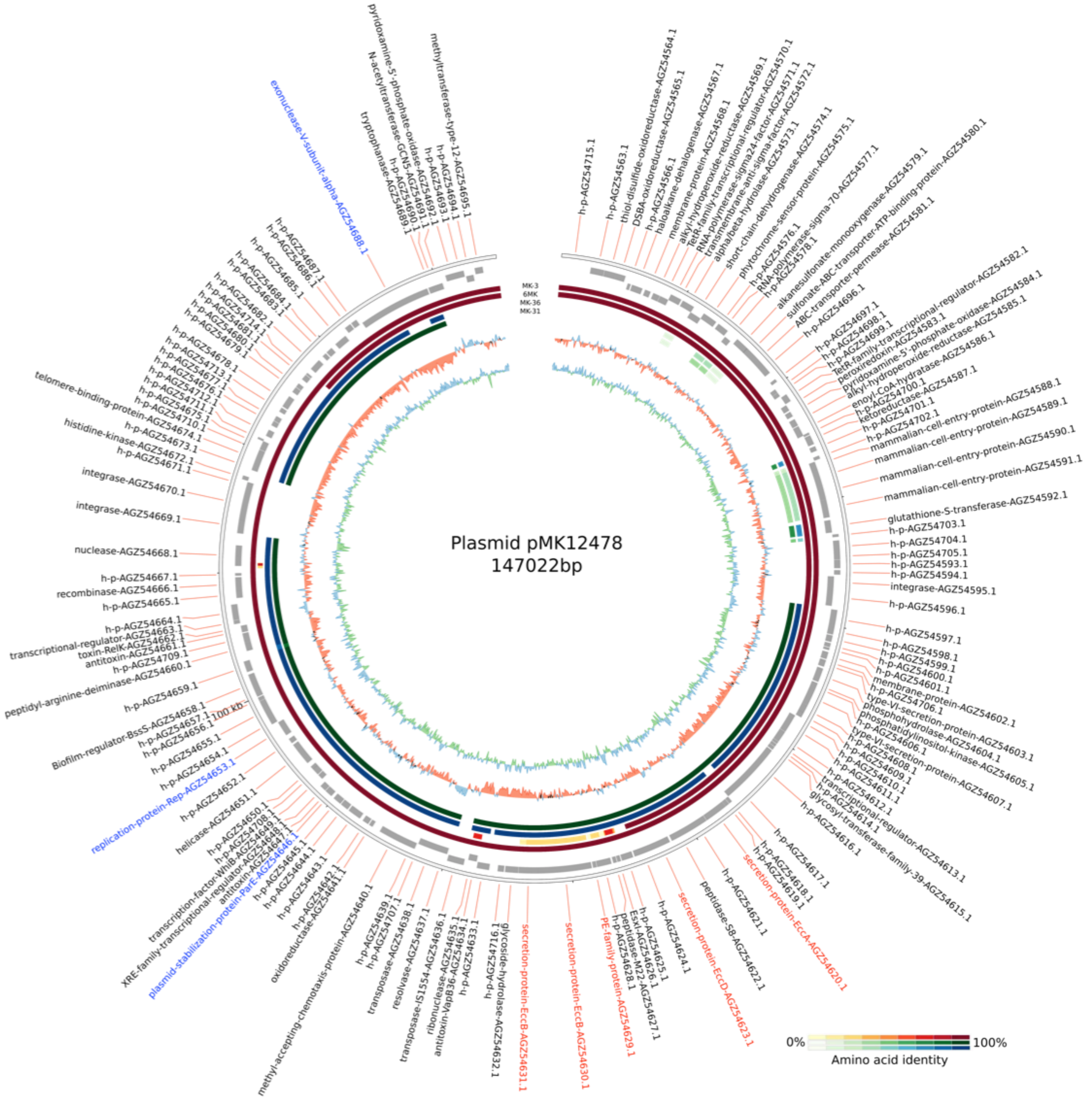

Figure S9 – Alignment of the complete plasmid sequence of *M. kansasii* strain ATCC 12478 and four closely related plasmids of the dataset.

The following features are displayed from in to out: GC content, GC skew, the PROmer (part of MUMmer v3.0) [79,80] alignments of *M. kansasii* strains MK11, MK36, 6MK and MK3 (in red, pathogenic, in blue non-pathogenic and in green, unknown), the open reading frames and finally the gene annotations. Type-IV and type-VII secretion system homologs are highlighted in blue and in red, respectively.

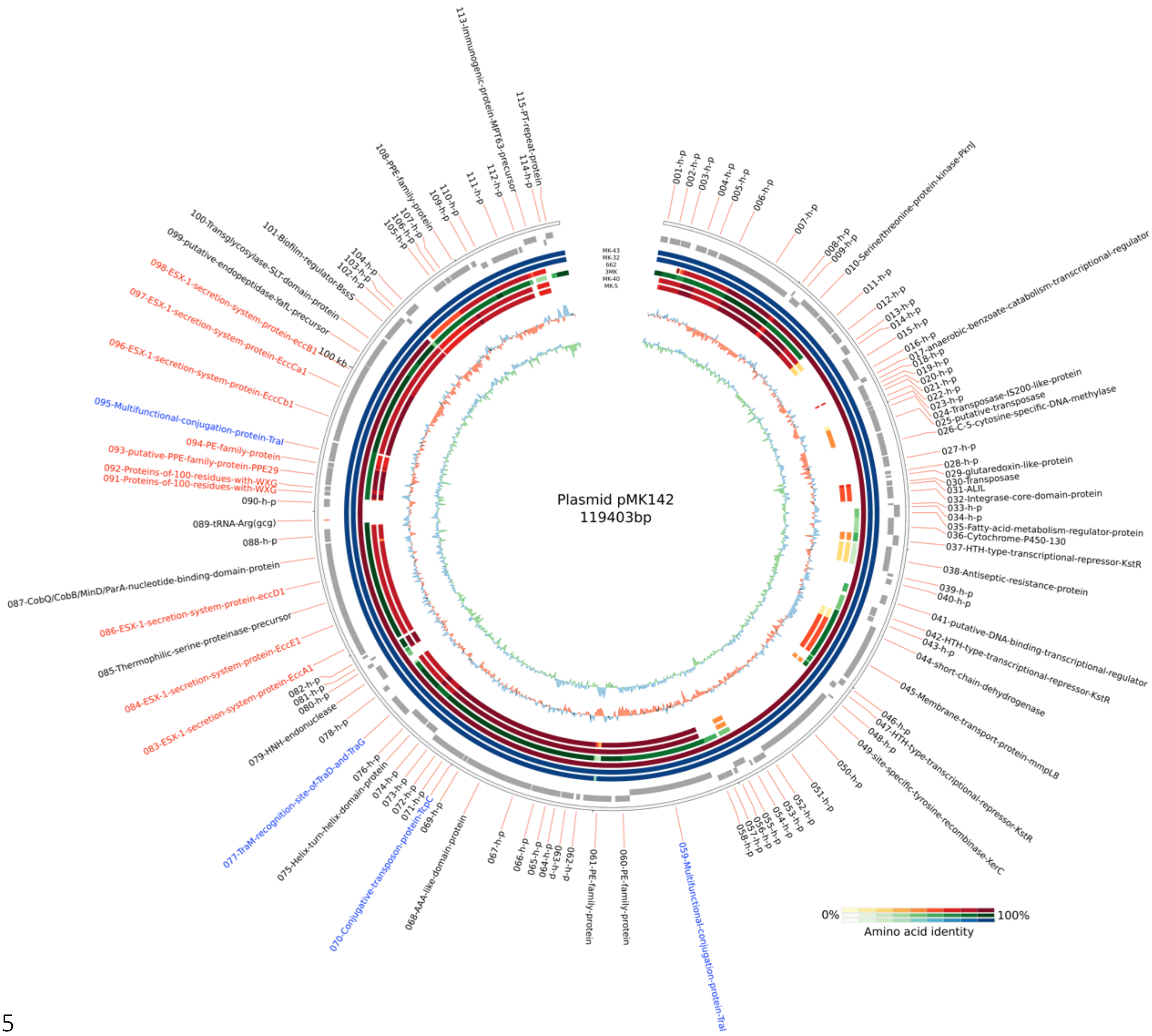

Figure S10 – Alignment of the complete plasmid sequence of *M. pseudokansasii* strain MK142 and six closely related plasmids of the dataset.

The following features are displayed from in to out: GC content, GC skew, the PROmer (part of MUMmer v3.0) [79,80] alignments of strains MK43, MK32, 662, 3MK, MK40 and MK5 (in red, *M. kansasii*; in blue, *M. pseudokansasii* and in green, *M. persicum*), the open reading frames and finally the gene annotations. Type-IV and type-VII secretion system homologs are highlighted in blue and in red, respectively.

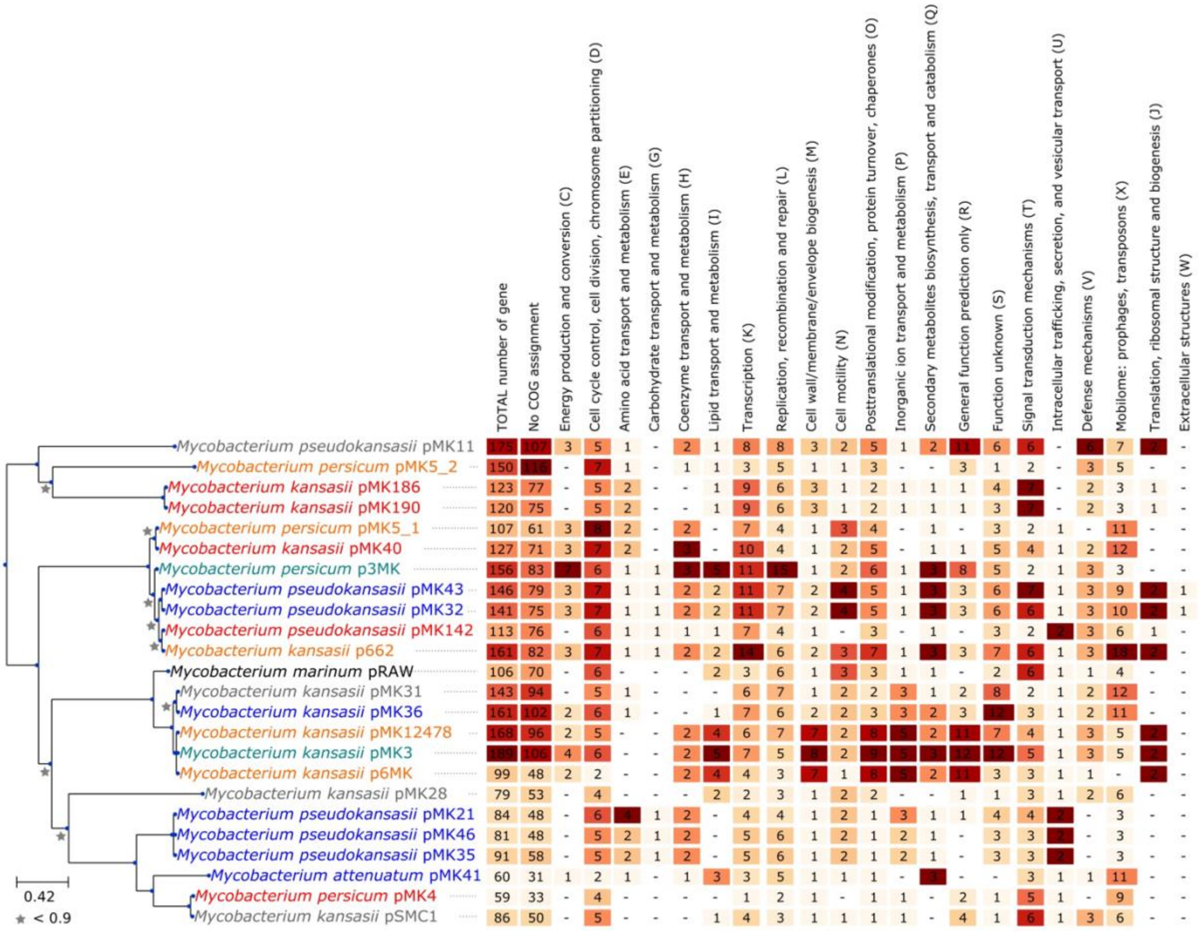

Figure S11 – Cluster of Orthologous Genes (COG) annotated on the identified conjugative plasmids. The maximum-likelihood is the same as presented in Figure 5B. The counts of each COG category found on the respective plasmids are indicated. The total count of annotated COG matched perfectly with plasmids shorter than 100Kb. Plasmid-encoded genes exhibited very diverse COG functions.

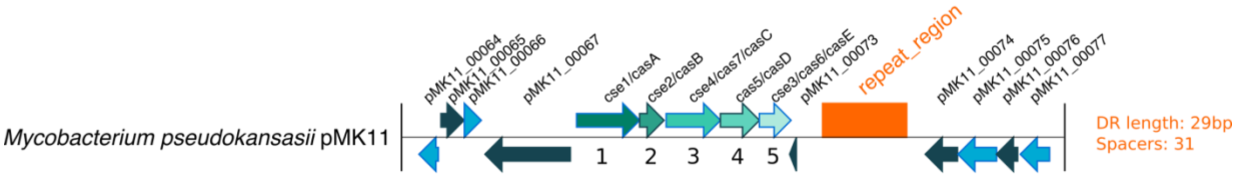

		qcov	e-value	ID
1. Hypothetical protein (cse1)	*Mycobacterium abscessus*	99%	0.0	65%
2. Hypothetical protein (cse2)	*Mycobacterium abscessus*	90%	4e-63	62%
3. Cas7/Cse4/CasC (Type I-E)	*Mycobacterium abscessus*	100%	0.0	72%
4. Cas5	*Mycobacterium abscessus*	92%	7e-86	59%
5. Hypothetical protein (cas6)	*Mycobacterium abscessus*	100%	1e-93	59%

Figure S12 – Plasmid-encoded CRISPR-cas system

Here is shown the locus encoding for a CRISPR-Cas system type-I-E genome that is encoded on the conjugative plasmid of *M. pseudokansasii* MK11. When BLASTing the sequences using BLASTP against the non-redundant protein database of the NCBI, best blast hits were Cas proteins of *Mycobacterium abscessus,* a fast-growing mycobacterium. This suggests that conjugative plasmids can be transferred between distantly related mycobacteria.
